# Atomically Precise Platinum Carbonyl Nanoclusters: Synthesis, Total Structure, and Electrochemical Investigation of [Pt_27_(CO)_31_]^4–^ Displaying a Defective Structure

**DOI:** 10.1021/acs.inorgchem.2c00965

**Published:** 2022-08-03

**Authors:** Cristiana Cesari, Beatrice Berti, Tiziana Funaioli, Cristina Femoni, Maria Carmela Iapalucci, Daniele Pontiroli, Giacomo Magnani, Mauro Riccò, Marco Bortoluzzi, Federico Maria Vivaldi, Stefano Zacchini

**Affiliations:** † Dipartimento di Chimica Industriale “Toso Montanari”, Università di Bologna, Viale Risorgimento 4, Bologna 40136, Italy; ‡ Dipartimento di Chimica e Chimica Industriale, Università di Pisa, Via G. Moruzzi 13, Pisa 56124, Italy; § Dipartimento di Scienze Matematiche, Fisiche e Informatiche, and INSTM, Università degli Studi di Parma, Viale delle Scienze 7/a, Parma 43124, Italy; ∥ Dipartimento di Scienze Molecolari e Nanosistemi, Ca’Foscari University of Venice, Via Torino 155, Mestre (Ve) 30175, Italy

## Abstract

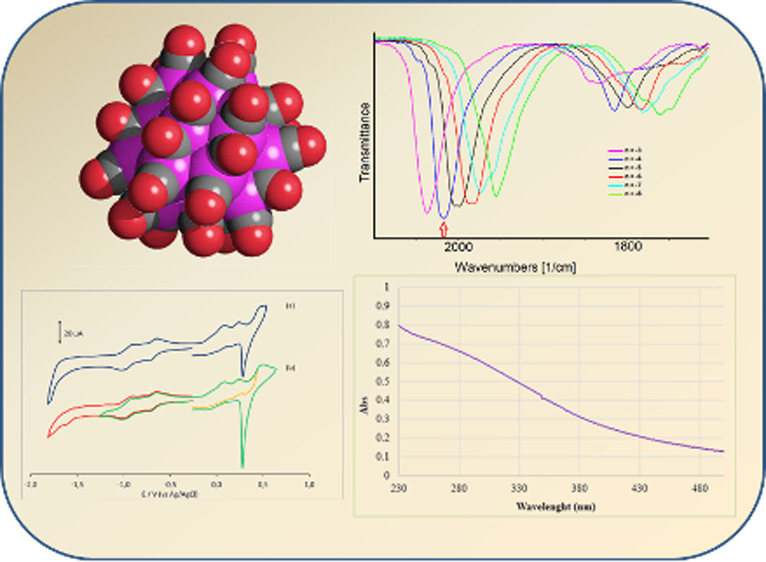

The molecular Pt nanocluster [Pt_27_(CO)_31_]^4–^ (**1**^**4–**^) was obtained by thermal decomposition of [Pt_15_(CO)_30_]^2–^ in tetrahydrofuran under a H_2_ atmosphere. The reaction of **1**^**4–**^ with increasing amounts of HBF_4_**·**Et_2_O afforded the previously reported [Pt_26_(CO)_32_]^2–^ (**3**^**2–**^) and [Pt_26_(CO)_32_]^−^ (**3**^**–**^). The new nanocluster **1**^**4–**^ was characterized by IR and UV–visible spectroscopy, single-crystal X-ray diffraction, direct-current superconducting quantum interference device magnetometry, cyclic voltammetry, IR spectroelectrochemistry (IR SEC), and electrochemical impedance spectroscopy. The cluster displays a cubic-close-packed Pt_27_ framework generated by the overlapping of four ABCA layers, composed of 3, 7, 11, and 6 atoms, respectively, that encapsulates a fully interstitial Pt_4_ tetrahedron. One Pt atom is missing within layer 3, and this defect (vacancy) generates local deformations within layers 2 and 3. These local deformations tend to repair the defect (missing atom) and increase the number of Pt–Pt bonding contacts, minimizing the total energy. The cluster **1**^**4–**^ is perfectly diamagnetic and displays a rich electrochemical behavior. Indeed, six different oxidation states have been characterized by IR SEC, unraveling the series of **1**^***n***–^ (*n* = 3–8) isostructural nanoclusters. Computational studies have been carried out to further support the interpretation of the experimental data.

## Abstract 2

The synthesis, molecular structure, magnetic properties, computational investigation, electrochemical and IR spectroelectrochemical behavior, and electrochemical impedance spectroscopy investigation of the new multivalent [Pt_27_(CO)_31_]^4−^ nanocluster are reported.

## 1 Introduction

Atomically precise metal nanoclusters have attracted impressive interest since the discovery of the thiolate-protected Au_102_.^1−5^ Most of the literature has been dedicated to molecular Au nanoclusters and then to Cu and Ag nanoclusters as well as related molecular alloy nanoclusters.^6−14^ The researches on atomically precise Pt nanoclusters have also grown, in view of the widespread use of Pt complexes, nanoparticles, and crystallites in catalysis and electrocatalysis.^15−20^ Gaining atomic precision on Pt nanoclusters dispersed on supports and electrode materials would offer the possibility of increasing our knowledge on the fundamental steps involved in catalytic and electrocatalytic processes.^21−26^ Moreover, the properties of such materials could be improved and finely tuned depending on the dimension and structure of the Pt nanoclusters.

Platinum carbonyl clusters represent the richest class of atomically precise Pt nanoclusters that have been structurally characterized to date.^27,28^ These can be classified based on their carbon monoxide (CO) content as (a) CO-rich clusters (CO/Pt = 2) of the type [Pt_3*n*_(CO)_6*n*_]^2–^ (*n* = 2–8), the so-called Chini clusters, and (b) CO-poorer species (CO/Pt < 2), which comprise globular molecular Pt nanoclusters, often referred to as Pt browns in view of the color of their solutions. Chini clusters adopt 1D structures based on triangular Pt_3_(CO)_3_(μ-CO)_3_ units stacked along a pseudo-*C*_3_ axis. Moreover, in the solid state, they can self-assemble into conductive Pt wires.^29−33^ Conversely, Pt browns so far characterized contain from 14 up to 44 metal atoms and possess more compact 3D structures such as pentagonal prismatic (pp), body-centered-cubic (bcc), cubic-close-packed (ccp), hexagonal-close-packed (hcp), or twinned hcp/ccp.^34−40^ It must be remarked that larger Pt nanoparticles as well as bulk Pt systematically adopt a ccp structure. This rich diversity of the structures of molecular Pt nanoclusters compared to larger nanoparticles is analogous to the case of atomically precise Au nanoclusters in comparison the larger Au nanoparticles. Thus, it seems to be a size effect independent of the nature of the metals and/or ligands.^41,42^

Globular platinum carbonyl nanoclusters often adopt highly symmetric regular structures, that is, [Pt_19_(CO)_22_]^4–^, [Pt_24_(CO)_30_]^2–^, [Pt_26_(CO)_32_]^*n*−^ (*n* = 1, 2), [Pt_38_(CO)_44_]^2–^, [Pt_40_(CO)_40_]^6–^, and [Pt_44_(CO)_45_]^*n*−^ (*n* = unknown).^34−40^ Nonetheless, [Pt_23_(CO)_27_]^2–^, [Pt_33_(CO)_38_]^2–^, and [Pt_36_(CO)_44_]^2–^ display less regular and defective structures, originating from the removal of one or more metal atoms from a compact structure.^34^ Systematically, deformations occur nearby such local defects in order to fix them. Similar localized phenomena may occur during heterogeneous catalysis employing ultradispersed metal nanoparticles, as shown in the case of Pt_12_ and Pt_13_ nanoclusters encapsulated within dendrimers employed for the oxygen reduction reaction.^24^

As a further point of interest, large molecular metal carbonyl clusters, including a few atomically precise platinum carbonyl nanoclusters,^34,36^ are multivalent, displaying several reversible or quasi-reversible redox processes. This behavior can be studied in detail by means of electrochemical methods as well as IR spectroelectrochemical (IR SEC) studies.^43,44^ The fact that molecular metal nanoclusters can exist with a variable number of electrons is a direct consequence of the incipient metalization of their metal core with increasing size.

In the present paper, the synthesis and total structure determination by single-crystal X-ray diffraction (SC-XRD) of the unprecedented atomically precise [Pt_27_(CO)_31_]^4–^ (**1**^**4–**^) nanocluster, displaying a defective ccp structure, is reported. The new nanocluster has been further characterized through IR and UV–visible spectroscopies and its diamagnetism ascertained by superconducting quantum interference device (SQUID) measurements. In addition, the redox properties of **1**^**4–**^ have been investigated by means of electrochemical and IR SEC methods, as well as electrochemical impedance spectroscopy (EIS). Computational studies of **1**^**4–**^ have been carried out to further support interpretation of the experimental data.

## 2 Experimental Section

### 2.1 General Procedures

All reactions and sample manipulations were carried out using standard Schlenk techniques under nitrogen and in dried solvents. All of the reagents were commercial products (Aldrich) of the highest purity available and used as received, except [PPh_4_]_2_[Pt_15_(CO)_30_], which has been prepared according to the literature.^45^ Analyses of C, H, and N were obtained with a Thermo Quest Flash EA 1112NC instrument. IR spectra were recorded on a PerkinElmer Spectrum One interferometer in CaF_2_ cells. Absorption spectra were recorded at 298 K using an Agilent Cary 100 UV–visible spectrometer. Structure drawings were obtained with *SCHAKAL99*.^46^

### 2.2 Synthesis of [PPh_4_]_4_[Pt_27_(CO)_31_] ([PPh_4_]_4_[**1**])

A solution of [PPh_4_]_2_[Pt_15_(CO)_30_] (0.700 g, 0.158 mmol) in tetrahydrofuran (THF; 20 mL) was heated at 65 °C under a H_2_ atmosphere for 8 h. Then, the solvent was removed under reduced pressure and the residue washed with H_2_O (3 × 15 mL), isopropyl alcohol (3 × 15 mL), toluene (3 × 15 mL), and THF (3 × 15 mL) and extracted in acetone (20 mL). Crystals of [PPh_4_]_4_[**1**]·CH_3_COCH_3_·solv suitable for SC-XRD were obtained by slow diffusion of isopropyl alcohol (50 mL) on the acetone solution (yield 0.32 g, 48% based on Pt).

Calcd for C_130_H_86_O_32_P_4_Pt_27_ (7551.29): C, 20.68; H, 1.15. Found: C, 20.38; H, 1.31. IR (Nujol, 293 K): ν_CO_ 2003(vs), 1953(sh), 1849(m), 1803(s), 1770(ms), 1747(ms) cm^–1^. IR (CH_3_CN, 293 K): ν_CO_ 2018(vs), 1814(m), 1777(w) cm^–1^. IR (acetone, 293 K): ν_CO_ 2018(vs), 1816(m) cm^–1^. IR (CH_2_Cl_2_, 293 K): ν_CO_ 2019(vs), 1811(m).

### 2.3 Synthesis of [PPh_4_][Pt_26_(CO)_32_] ([PPh_4_][**3**])

HBF_4_·Et_2_O (15 μL, 0.110 mmol) was added to a solution of [PPh_4_]_4_[**1**] (0.354 g, 0.047 mmol) in acetone (15 mL) up to the formation of **3**^**2–**^, as indicated by IR spectroscopy [ν_CO_ 2044(vs), 1803(m) cm^–1^]. The solvent was removed under reduced pressure, the residue was dissolved in CH_2_Cl_2_ (15 mL), and further HBF_4_·Et_2_O (15 μL, 0.110 mmol) was added up to the formation of **3**^**–**^ [ν_CO_ 2062(vs), 1820(m) cm^–1^]. The resulting solution was filtered and layered with *n*-hexane (30 mL), resulting in crystals of [PPh_4_][**3**] suitable for SC-XRD (yield 0.16 g, 51% based on Pt).

Calcd for C_56_H_20_O_32_PPt_26_ (6308.03): C, 10.66; H, 0.32. Found: C, 10.89; H, 0.57. IR (CH_2_Cl_2_, 293 K): ν_CO_ 2062(vs), 1820(m) cm^–1^.

### 2.4 X-ray Crystallographic Study

The crystal data and collection details for [PPh_4_]_4_[**1**]·CH_3_COCH_3_·solv and [PPh_4_][**3**] are reported in Table S1. The diffraction experiments were carried out on a Bruker APEX II diffractometer equipped with a PHOTON2 detector using Mo Kα radiation. Data were corrected for Lorentz polarization and absorption effects (empirical absorption correction *SADABS*).^47^ Structures were solved by direct methods and refined by full-matrix least squares based on all data using *F*^2^.^48^ H atoms were fixed at calculated positions and refined by a riding model. All non-H atoms were refined with anisotropic displacement parameters, unless otherwise stated.

#### [PPh_4_]_4_[**1**]·CH_3_COCH_3_·solv

The asymmetric unit of the unit cell contains one cluster anion, four [PPh_4_]^+^ cations, and one CH_3_COCH_3_ molecule, all located on general positions. The unit cell contains an additional total potential solvent-accessible void of 1155 Å^3^ (ca. 8% of the cell volume), which is likely to be occupied by highly disordered solvent molecules. These voids have been treated using the *SQUEEZE* routine of *PLATON*.^49,50^ The C and O atoms of the CO ligands in the cluster anion and the acetone molecule were restrained to isotropic behavior (the ISOR line in *SHELXL*, s.u. 0.01). Similar *U* restraints were applied to the C and P atoms of the [PPh_4_]^+^ cation and to the C and O atoms of the acetone molecule (SIMU line in SHELXL, s.u. 0.01). The C atoms of the aromatic rings were constrained to fit regular hexagons (the AFIX 66 line in *SHELXL*). Restraints to bond distances were applied as follows (s.u. 0.02): 1.21 Å for C–O and 1.51 Å for C–C in CH_3_COCH_3_.

#### [PPh_4_][**3**]

The asymmetric unit of the unit cell contains one cluster anion and one [PPh_4_]^+^ cation, all located on general positions. The C and O atoms of the CO ligands in the cluster anion were restrained to isotropic behavior (the ISOR line in *SHELXL*, s.u. 0.01). The [PPh_4_]^+^ cation is disordered, and, therefore, it was split into two positions and refined anysotropically by applying a rigid group refinement (the AFIX 6 line in *SHLEXL*). Similar *U* restraints were applied to the C and P atoms of the [PPh_4_]^+^ cation (the SIMU line in *SHELXL*, s.u. 0.01).

### 2.5 Magnetic Measurements

Magnetic susceptibility measurements were performed on powder samples with a Quantum Design MPMS XL SQUID magnetometer, capable of fields as high as 5 T. The samples for SQUID measurements were handled in an Ar glovebox (<1 ppm of H_2_O, O_2_) and sealed in quartz ampules of 5 mm diameter filled with a low He pressure (approximately 5 Torr).

### 2.6 Electrochemical, Spectroelectrochemical, and EIS Measurements

Materials and apparatuses for electrochemistry and IR SEC have been described elsewhere.^43^ EIS spectra were recorded using as *E*_dc_ the *E*°′ of a reversible electrochemical reaction obtained from the voltammetric experiments. *E*_ac_ was set to 0.005 V, and the frequency was scanned between 10000 and 1 Hz. All of the electrochemical experiments were performed using a Palmsens 4 potentiostat (Palmsens, The Netherlands).

### 2.7 Computational Details

Geometry optimizations of the clusters and IR simulations were performed using the semiempirical tight-binding GFN2-xTB method^51^ in the gas phase and in the presence of acetone as a continuous medium thanks to the addition of an analytical linearized Poisson–Boltzmann (ALPB) solvation model.^52^ Further calculations were carried out in the gas phase using the PBEh-3c method, which is a reparametrized version of PBE0 (with 42% HF exchange) that uses a split-valence double-ζ basis set (def2-mSVP) and adds three corrections that consider dispersion, basis set superposition, and other basis set incompleteness effects.^53^ The “restricted” approach was used in all cases.

The software used for GFN2-xTB calculations was *XTB*, version 6.5.0.^54^ PBEh-3c calculations were carried out with *ORCA*, version 5.0.3.^55^ The output, converted into .molden format, was elaborated with the software *Multiwfn*, version 3.5.^56^ Cartesian coordinates of the optimized structures are provided as separate .xyz files. IR spectra were plotted using *Gabedit*, version 2.5.1.^57^

## 3 Results and Discussion

### 3.1 Synthesis, Molecular (Total) Structure, Magnetic Behavior, and Reactivity of **1**^**4–**^

**Figure 1:**
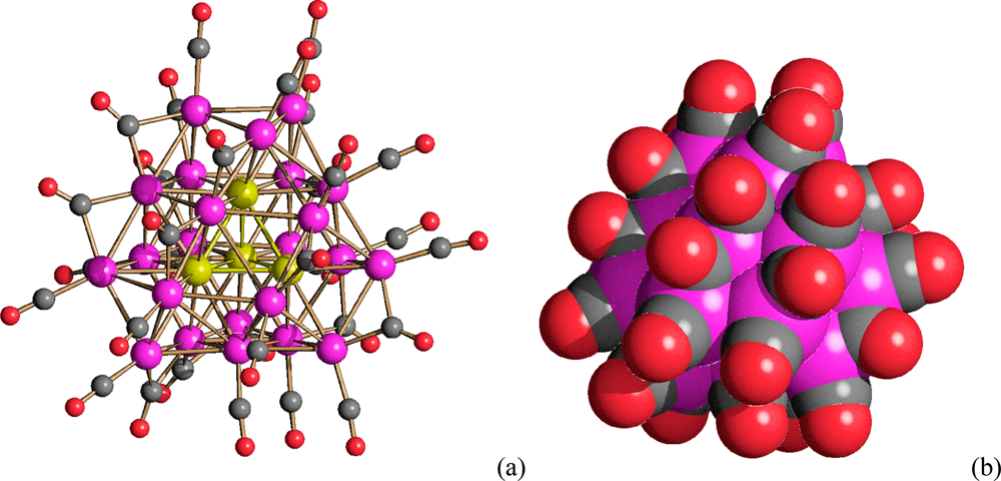
(a) Molecular structure of **1**^**4–**^ and (b) its space-filling model. Color code: purple, superficial Pt; yellow, interstitial Pt; red, O; gray, C. Pt–Pt bond distances: 2.633(3)–3.191(4) Å. Average distance: 2.81(3) Å.

**1**^**4–**^ was obtained as an oily precipitate after refluxing [Pt_15_(CO)_30_]^2–^ as [PPh_4_]^+^ salt in THF under a H_2_ atmosphere for 8 h. The formation of **1**^**4–**^ was accompanied by some [Pt_24_(CO)_30_]^2–^ (**2**^**2–**^), and the two clusters were separated by extraction with solvents of different polarity. Thus, **2**^**2–**^ was extracted in THF and **1**^**4–**^ in acetone. The previously known cluster **2**^**2–**^ was spectroscopically identified by a comparison with the published IR data.^36,38,39^ The molecular structure of **1**^**4–**^ was established by SC-XRD on its salt [PPh_4_]_4_[**1**]·CH_3_COCH_3_·solv (Figure 1). The quality of the crystal was low and the diffraction pattern at high 2θ very weak. Thus, the data were cut at 2θ = 48°. Despite the poor data quality, the overall structure of the cluster, including the metal cage and CO ligands, was well established, whereas some care must be taken in discussing the bonding distances involving lighter atoms.

The ^1^H NMR spectra of **1**^**4–**^ recorded overnight in both deuterated acetonitrile (CH_3_CN) and acetone at room temperature did not display any resonance in the hydride region. A large chemical shift window was employed (from +50 and −100 ppm), and delays from 1 up to 20 s were employed to compensate for potential relaxation problems. The possibility that the detection of hydrides at room temperature failed because of fluxionality was then checked by performing ^1^H NMR spectroscopy at lower temperatures (down to −80 °C) under similar experimental conditions. Also, these experiments did not show any resonances attributable to potential hydrides.

**Figure 2:**
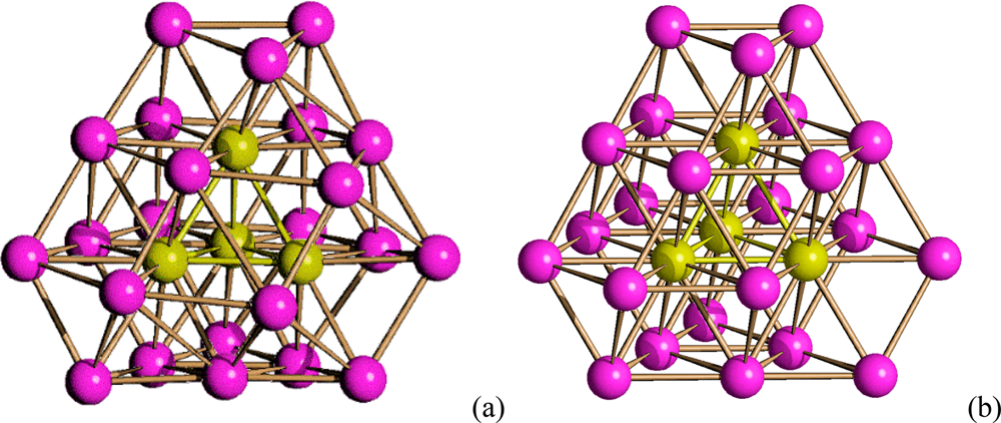
(a) Pt_27_ metal core of **1**^**4–**^ containing a fully interstitial Pt_4_ tetrahedron compared to (b) an idealized Pt_27_ ccp fragment. Color code: purple, superficial Pt; yellow, interstitial Pt.

The molecular structure of **1**^**4–**^ is composed of a ccp Pt_27_ framework generated by the overlapping of four ABCA layers, composed of 3, 7, 11, and 6 atoms, respectively. As shown in Figure 2, this structure contains a fully interstitial Pt_4_ tetrahedron. The structure is completed by 18 terminal and 13 edge-bridging carbonyls. In agreement with this, the IR spectrum in an acetonitrile (CH_3_CN) solution of **1**^**4–**^ displays ν_CO_ bands in the terminal (2018 cm^–1^) and bridging (1814 and 1777 cm^–1^) region.

**Figure 3:**
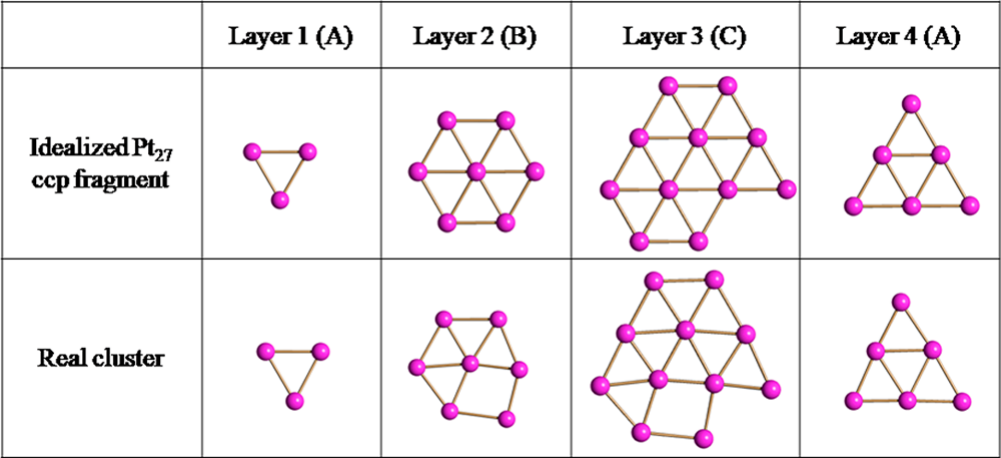
Four ABCA layers along (111) of the Pt_27_ metal core of **1**^**4–**^ compared to an idealized Pt_27_ ccp fragment.

One Pt atom is missing within layer 3 (Figure 3), which consists of 11 instead of 12 Pt atoms as expected for a compact layer. As a result of this defect (vacancy), local deformations are present within layers 2 and 3, whereas layers 1 and 4 are very close to the idealized ones. As a result of these deformations that occur in correspondence with the defect (missing atom), the number of the Pt–Pt bonding contacts increases from 91 in the idealized fragment to 93 in the real cluster. Moreover, the inner Pt_4_ tetrahedron, which is semiexposed in the idealized Pt_27_ ccp fragment, becomes fully interstitial in the idealized cluster.

**Figure 4:**
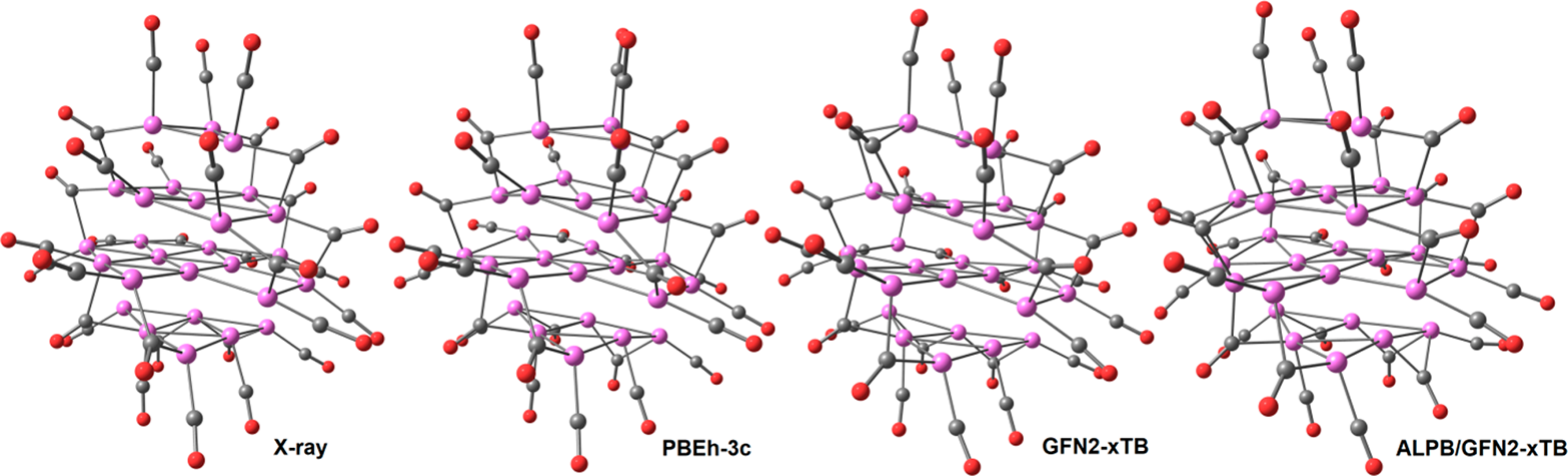
Comparison of the experimental and computationally optimized structures of **1**^**4–**^. Color code: purple, Pt; red, O; gray, C. The Pt–Pt interactions among the ABCA layers were not drawn for clarity.

The structure of **1**^**4–**^ was computationally investigated by means of the PBEh-3c and GFN2-xTB methods, the latter also in combination with the ALPB implicit solvation model (further details are reported in the Experimental Section). All of the approaches afforded stationary points in line with the experimental data, with a root-mean-square deviation (RMSD) between the computed and experimental geometries of 0.520 Å (PBEh-3c), 0.485 Å (GFN2-xTB), and 0.593 Å (ALPB/GFN2-xTB). The RMSD values were even smaller upon a comparison of only the {Pt_27_} cores, with values equal to 0.243 Å (PBEh-3c), 0.233 Å (GFN2-xTB), and 0.280 Å (ALPB/GFN2-xTB). The arrangement of the Pt atoms in **1**^**4–**^ appears therefore to be indirectly confirmed by density functional theory (DFT) calculations, as is also observable in Figure 4. Superimpositions of the X-ray and PBEh-3c structures highlighting the good overlap between the experimental and computed geometries, in particular for those concerning the {Pt_27_} core, are shown in Figure S1.

The possible presence of hydrides not experimentally detected in **1**^**4–**^ was computationally explored, maintaining constant the global charge and even number of electrons. Starting from several different initial geometries, attempts to optimize clusters having the formula [Pt_27_H_2_(CO)_31_]^4–^ at the GFN2-xTB and PBEh-3c levels afforded species with terminal hydrides as the most stable isomers (Figure S2). A comparison with the experimental structure of **1**^**4–**^ did not evidence excessive distortions, as indicated by the RMSD values reported in the caption of Figure S2. Moreover, the formation of a dihydride is thermodynamically very unlikely because the energy variation for the reaction **1**^**4–**^ + H_2_ → [Pt_27_H_2_(CO)_31_]^4–^ is meaningfully positive, 43.9 kcal mol^–1^ (PBEh-3c calculations, with the energy variation calculated from the sums of the corrected electronic energies and nuclear repulsions).

**Figure 5:**
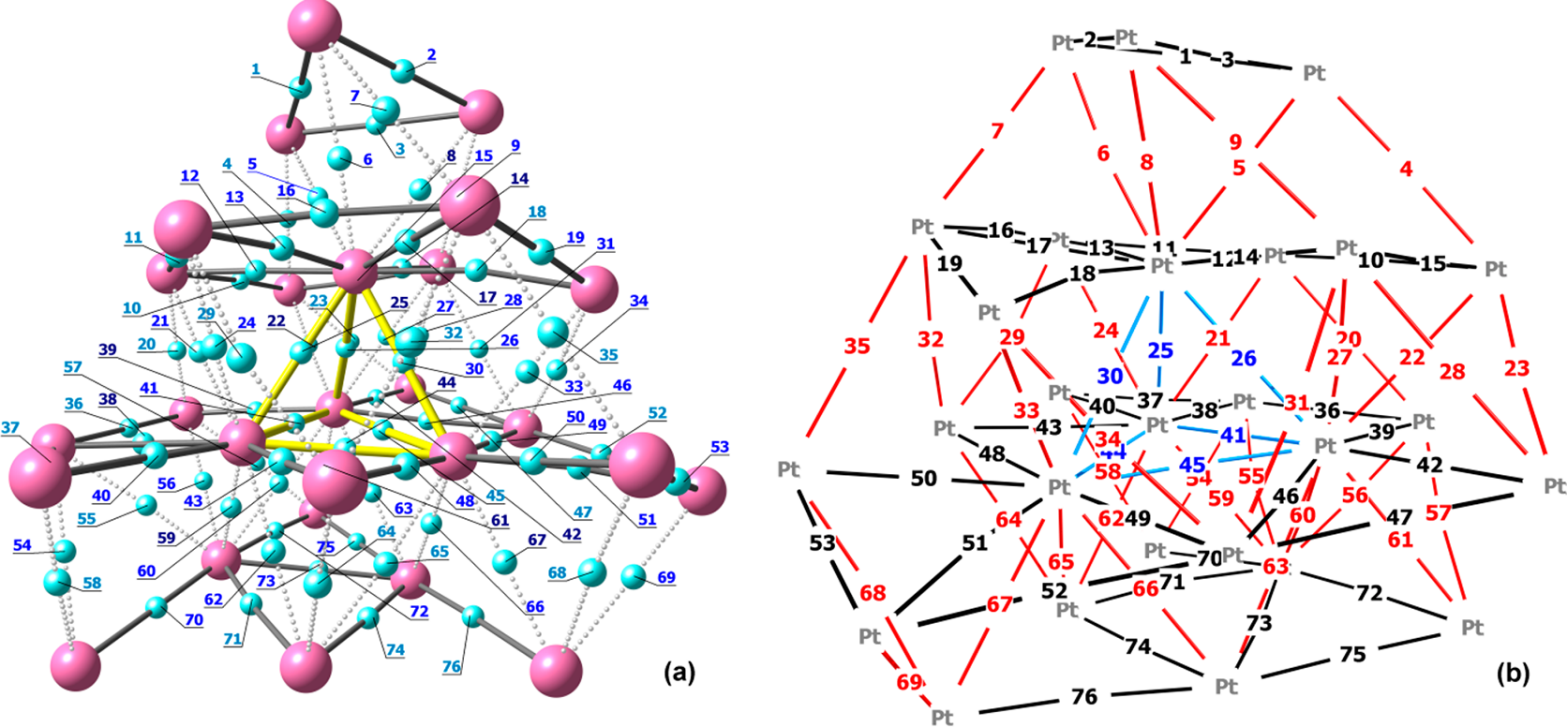
Two views of the Pt–Pt (3, -1) b.c.p.’s with numbering (PBEh-3c calculations). In part a, the Pt atoms are in purple and the b.c.p.’s in cyan. The intralayer Pt–b.c.p.–Pt connections are represented with solid lines, the interlayer Pt–b.c.p.–Pt connections with dotted lines, and those in the interstitial {Pt_4_} tetrahedron as yellow lines. The b.c.p. numbers are colored accordingly to the ρ values: <0.270 e Å^–3^, light blue; between 0.270 and 0.400 e Å^–3^, blue; >0.400 e Å^–3^, dark blue. In part b, the intralayer Pt–(b.c.p.)–Pt connections are colored in black, the interlayer Pt–(b.c.p.)–Pt connections in red, and those in the interstitial {Pt_4_} tetrahedron in blue.

The “atoms in molecules” (AIM) analysis on the structure of **1**^**4–**^ optimized at the PBEh-3c level afforded 76 (3, −1) Pt–Pt bond critical points (b.c.p.’s), represented in Figure 5. Selected data regarding the b.c.p.’s are reported in Table S2. The negative values of the energy density (*E*) and the positive values for the Laplacian of the electron density (∇^2^ρ) are in agreement with Bianchi’s definition of the M–M bonds.^58,59^ The Hirshfeld charges^60^ on Pt atoms are comprised between −0.16 and +0.06 au. The four more negative values, from −0.16 to −0.14 au, are localized on the Pt atoms constituting the interstitial tetrahedron. The electron density at b.c.p. (ρ) ranges from 0.155 to 0.472 e Å^–3^, and the lower values were generally found in the outer region of the metal core. Accordingly, some Pt–Pt b.c.p.’s at the surface of {Pt_27_} were not localized, probably because of the competition of the carbonyl ligands in the localization of the bonds. The potential energy density (*V*) values at b.c.p. are roughly correlated to the electron density, with more negative values found for higher ρ. The *V* interval is between −0.115 and −0.493 hartree Å^–3^. The weakest Pt–Pt interaction corresponds to b.c.p. of 10, while the strongest ones are associated with b.c.p.’s of 38, 44 and 59.

The AIM data reported for **1**^**4–**^ were compared with those obtained for the smaller carbonyl cluster [Pt_14_(CO)_18_]^4–^^35^ optimized at the same theoretical level. The DFT-optimized structure is shown in Figure S3, and the data related to the Pt–Pt (3, −1) b.c.p.’s are summarized in Table S3. The two clusters have quite similar values, even if the ρ interval in [Pt_14_(CO)_18_]^4–^ is in the 0.193–0.444 e Å^–3^ range, more limited with respect to **1**^**4–**^. The same consideration is valid for the *V* values in [Pt_14_(CO)_18_]^4–^, comprised between −0.144 and −0.423 hartree Å^–3^. As for **1**^**4–**^, some Pt–Pt b.c.p.’s at the surface of the {Pt_14_} core of [Pt_14_(CO)_18_]^4–^ were not localized because of the dominating interactions with the CO ligands. The smaller size of [Pt_14_(CO)_18_]^4–^ does not allow one to appreciate the increase of ρ at Pt–Pt b.c.p. moving toward the center of the cluster previously described for **1**^**4–**^.

The simulations of the IR spectrum of **1**^**4–**^ gave as a result 31 active transitions (Table S4) related to the stretchings of the carbonyl ligands, according to the low symmetry of the metal cluster. The Gaussian line-shape fitting shown in Figure S4, however, afforded simulated spectra qualitatively comparable with the experimental one.

**Figure 6:**
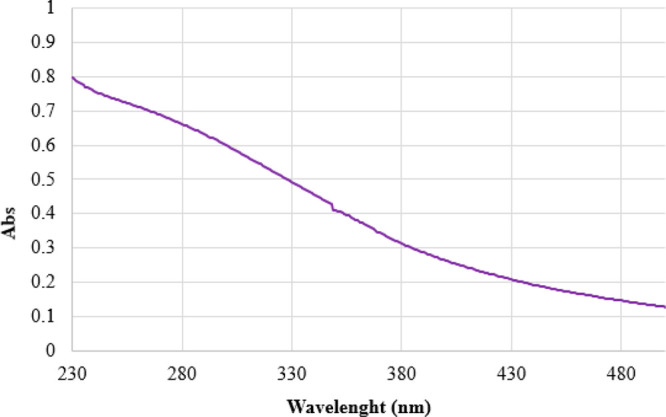
UV–visible absorption spectrum of **1**^**4–**^ in CH_3_CN at 298 K.

The UV–visible spectrum of **1**^**4–**^ in a CH_3_CN solution shows a featureless spectrum with a continuous and broad electronic absorption typical of interband transitions.^61^ Thus, the UV–visible spectrum of **1**^**4–**^ shown in Figure 6 closely resembles those of ultrasmall metal nanoparticles.

**Figure 7:**
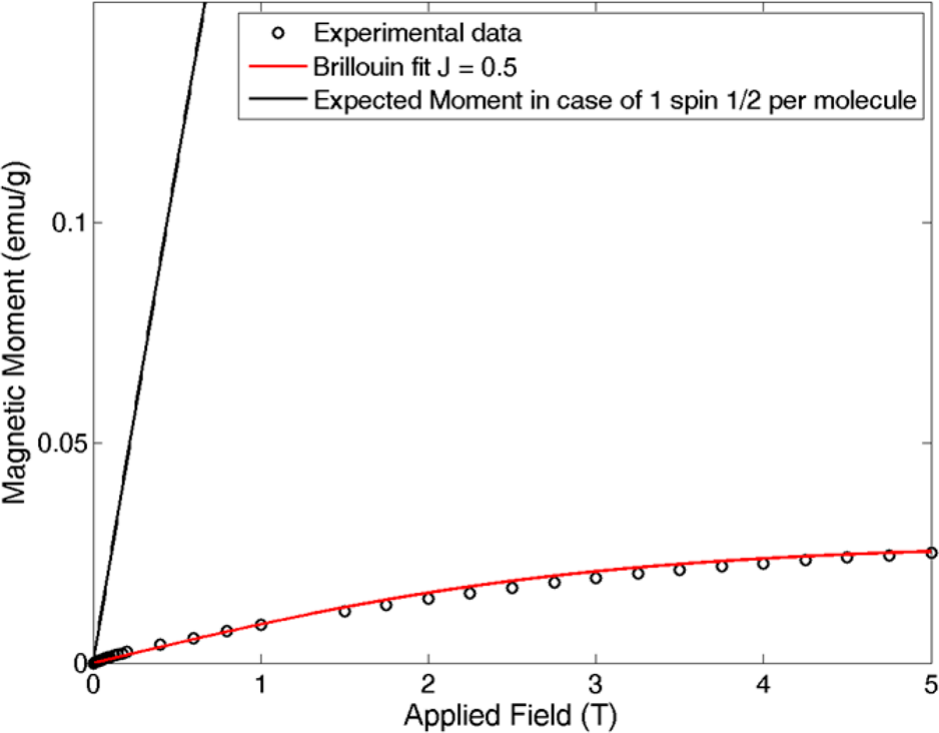
First magnetization curve for [PPh_4_]_4_[**1**] powder salt at 2 K. Data are displayed as black circles, while the solid red line represents the best fit with the Brillouin curve with *J* = 0.5. The small signal is compatible with the presence of paramagnetic impurities. The black line indicates the expected magnetic signal if each cluster would bear a spin ^1^/_2_.

The magnetic behavior of **1**^**4–**^ was investigated by performing dc SQUID magnetometry on the samples that were measured in the form of powder. A magnetization curve of the sample *M*(*H*) was collected at low temperature (2 K). The first magnetization curve is displayed in Figure 7 and is compatible with a paramagnetic behavior of the sample, with *J* = 0.5. However, the fit of the experimental data with the Brillouin curve indicated that such a paramagnetic signal would correspond to just 0.04 spin ^1^/_2_ per molecule, a very small magnetic fraction that likely is compatible with the presence of impurities, rather than intrinsic magnetism of the cluster, thus strongly suggesting that the sample is essentially diamagnetic. A much more intense signal would be expected if each molecule would bear a net magnetic moment (Figure 7).

**Scheme 1:**
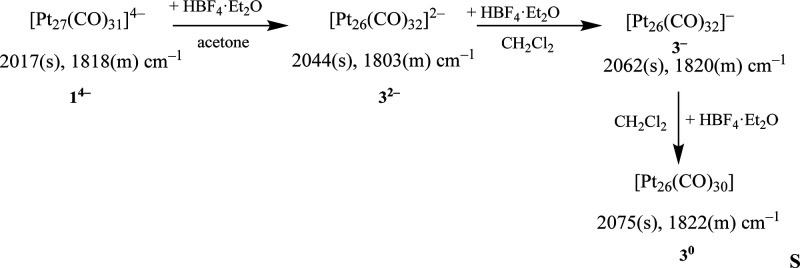
Reactions of **1**^**4–**^ with Acids, and Synthesis of **3**^***n*–**^ (*n* = 0, 1, 2)

**Figure 8:**
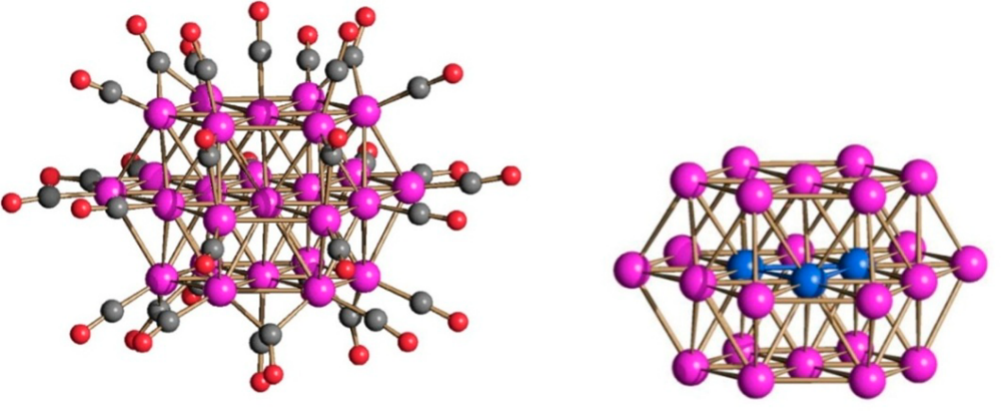
Molecular structure of **3**^**–**^. Color code: purple, Pt; red, O; gray, C; blue, interstitial Pt. Pt–Pd bond distances: 2.641(2)–2.9391(2) Å. Average distance: 2.793(19) Å.

The reaction of **1**^**4–**^ with increasing amounts of HBF_4_·Et_2_O afforded [Pt_26_(CO)_32_]^2–^ (**3**^**2–**^) and then [Pt_26_(CO)_32_]^−^ (**3**^**–**^) (Scheme 1). **3**^**2–**^ and **3**^**–**^ were identified by IR spectroscopy;^35,39^ moreover, the structure of **3**^**–**^ was determined by SC-XRD on its [PPh_4_][**3**] salt (Figure 8). The structure of **3**^**–**^ closely resembles that previously reported for the same monoanion with different cations as well as that of the dianion **3**^**2–**^. They are all based on a hcp ABA structure, composed of three layers of 7, 12, and 7 Pt atoms.

The only side product detected along the oxidation of **1**^**4–**^ to **3**^**2–**^ was some Pt metal. This is in agreement with the fact that **1**^**4–**^ contained 27 Pt atoms and **3**^**2–**^ only 26 Pt atoms. Partial degradation of the oxidized cluster should be the source of the additional CO ligand present in **3**^**2–**^ (32 CO ligands) compared to **1**^**4–**^ (31 CO ligands).

Further oxidation of **3**^**–**^ using an excess of HBF_4_·Et_2_O led to a purported neutral [Pt_26_(CO)_30_] (**3**^**0**^) cluster, as evidenced by IR spectroscopy. Because of its scarce stability, all attempts to crystallize **3**^**0**^ failed.

### 3.2 Electrochemistry and IR SEC

**Figure 9:**
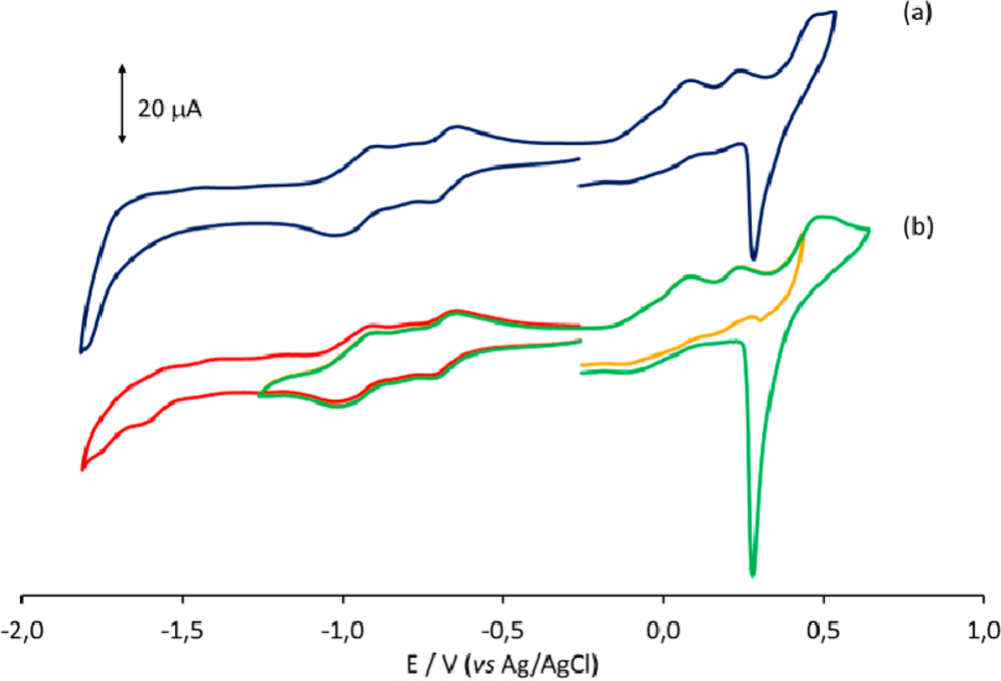
CV profiles recorded at the (a) Pt and (b) GC electrodes in a CH_3_CN solution of **1**^**4–**^. [N^*n*^Bu_4_][PF_6_] (0.1 mol dm^–3^) is the supporting electrolyte. Scan rate: 0.1 V s^–1^.

The redox behavior of **1**^**4–**^ in a CH_3_CN/[N^*n*^Bu_4_][PF_6_] solution was investigated on Pt and glassy carbon (GC) working electrodes (Figure 9). The same cyclic voltammetry (CV) profile was obtained on both electrodes, except at very low potentials, where two reductions were observable at the GC working electrode, while at the Pt electrode, the current increased without well-defined peaks. Only the two redox processes at −0.67 and −0.96 V (vs Ag/AgCl, KCl sat.) are resolved and appear to be chemically reversible. In the anodic region, at least three processes are present, whose chemical reversibility does not appear to be complete. Moreover, an intense peak, due to adsorption of the electrogenerated species, is present in the back-scan after the more anodic process.

**Figure 10:**
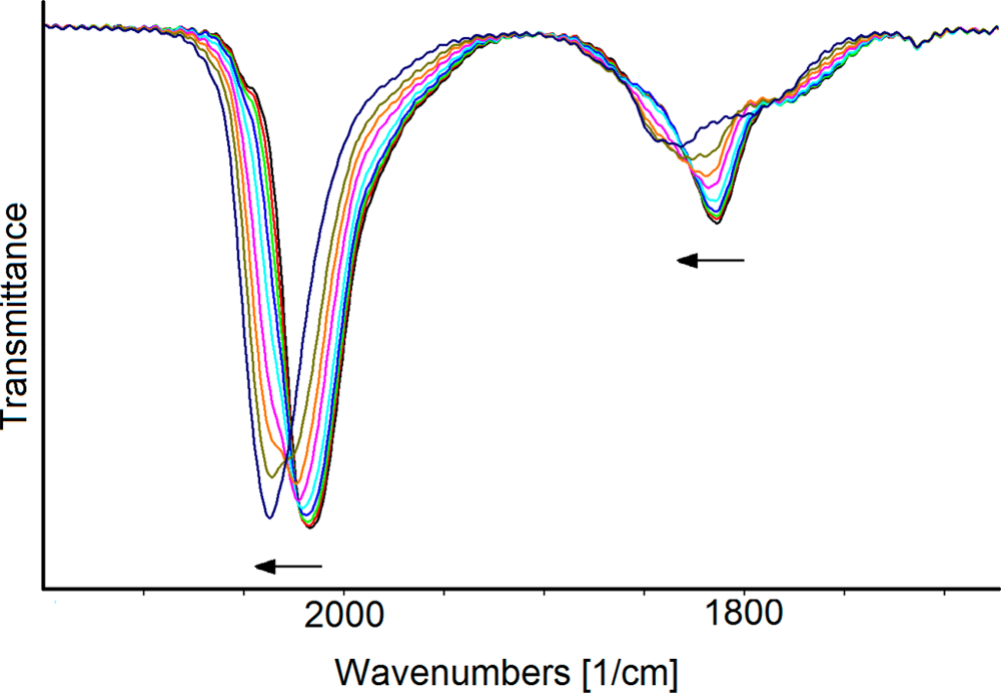
IR spectral changes of a CH_3_CN solution of **1**^**4–**^ recorded in an OTTLE cell during the progressive increase of the potential from −0.24 to +0.24 V versus Ag pseudoreference electrode (scan rate: 1 mV s^–1^). [N^*n*^Bu_4_][PF_6_] (0.1 mol dm^–3^) is the supporting electrolyte. The absorptions of the solvent and supporting electrolyte have been subtracted.

The redox processes of **1**^**4–**^ were studied by *in situ* IR SEC experiments conducted in an optical transparent thin-layer electrochemical (OTTLE) cell.^62^ IR spectra were measured at 60 s intervals during the slow scan (1 mV s^–1^) between selected potential values (vs Ag pseudoreference electrode). When the potential was raised from −0.24 to +0.30 V (vs Ag pseudoreference electrode), the CO stretching frequencies of **1**^**4–**^ (2018, 1814, and 1777 cm^–1^) shifted toward higher values (2037, 1832, and 1797 cm^–1^; Figure 10). This was attributed to formation of the oxidized **1**^**3–**^.

The shift of the IR bands at higher wavenumbers was correctly predicted for the simulated structure of **1**^**3–**^ (one unpaired electron) using semiempirical tight-binding approaches, as is observable in Table S5 and Figure S4. The optimized geometries of **1**^**3–**^ closely resemble those calculated for the corresponding **1**^**4–**^ cluster. The RMSD between **1**^**3–**^ and **1**^**4–**^ at the GFN2-xTB level is 0.362 Å (0.157 Å if considering only the {Pt_27_} fragment), and the main variation is related to the coordination mode of one carbonyl, from terminal in **1**^**4–**^ to bridging in **1**^**3–**^. The ALPB/GFN2-xTB-optimized structures of **1**^**3–**^ and **1**^**4–**^ are even closer (RSMD of 0.096 Å; 0.045 Å if considering only the metal core). The optimized structures of **1**^**3–**^ are shown in Figure S5.

Fairly defined isosbestic points were maintained during the formation of **1**^**3–**^, which appeared complete at +0.24 V. However, in the time elapsed at the higher potential, during the inversion of the scan direction, relatively fast decomposition of **1**^**3–**^ was pointed out by a sudden reversal of the ν^b^_CO_ shift direction (from 1832 to 1801 cm^–1^) accompanying a further upper shift of that related to the terminal ones (from 2037 to 2045 cm^–1^; Figure S6).

At the end of the reverse reduction back-scan, in the IR recorded at the initial potential, the band at 1814 cm^–1^ of the starting cluster was not quantitatively restored (Figure S7) and a new absorption arose at 1769 cm^–1^, while the ν^t^_CO_ maximum was at 2020 cm^–1^. These observations are in accordance with the formation of **3**^**2–**^ (2048 and 1803 cm^–1^) as a product of partial decomposition of the electrogenerated **1**^**3–**^ and its reduction to [Pt_26_(CO)_32_]^4–^ (**3**^**4–**^) (2020 and 1768 cm^–1^) in the reverse back-scan.^39^ Our conclusions were confirmed by the chemical oxidation of **1**^**4–**^ with increasing amounts of HBF_4_·Et_2_O, which quantitatively resulted in **3**^**2–**^ (see above).

To further clarify the voltammetric profile observed in the anodic region of Figure 9, we performed an IR SEC oxidation of **1**^**4*****–***^ from −0.24 to +0.80 V (Figure S8). The shift of ν^t^_CO_ up to 2048 cm^–1^ and a single absorption at 1803 cm^–1^ for bridging carbonyl groups indicated the complete formation of **3**^**2–**^, while a progressive decrease of the intensity of all of the carbonyl absorptions at increasing potentials pointed out the formation of an insoluble oxidized cluster.

**Figure 11:**
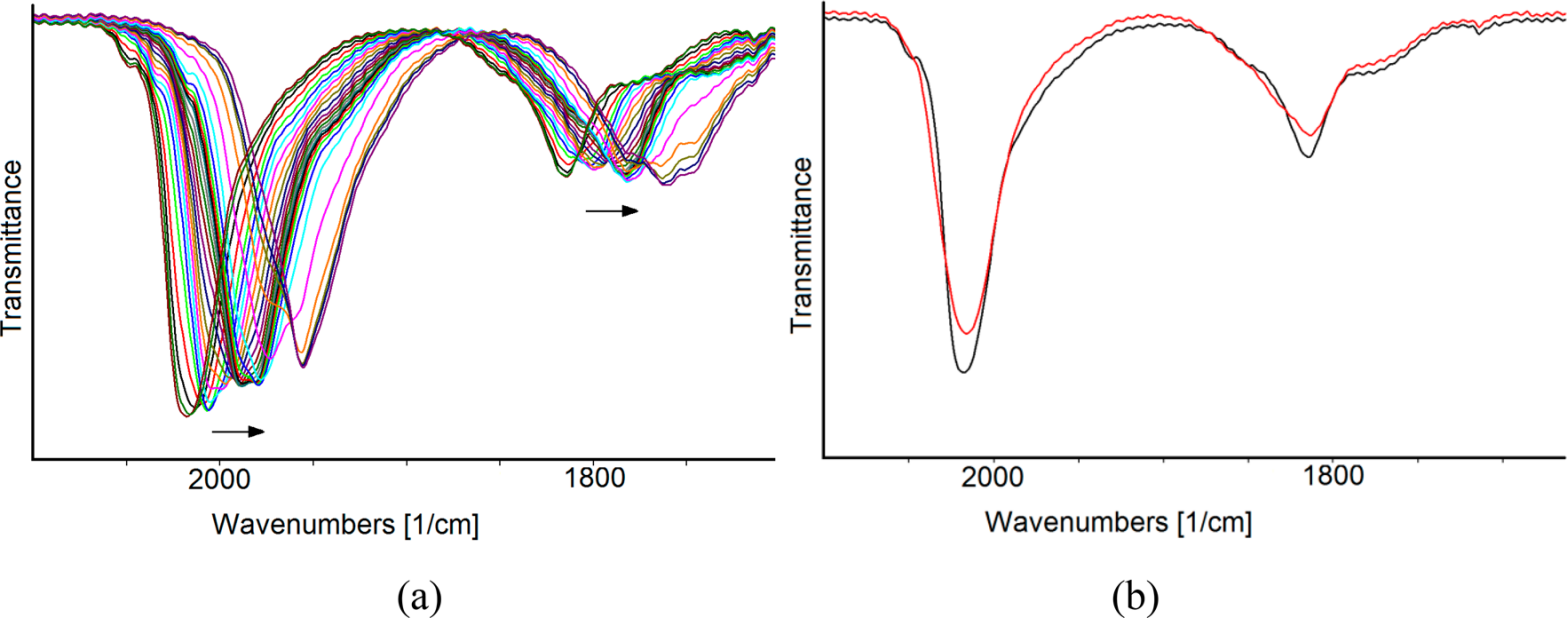
IR spectra of a CH_3_CN solution of **1**^**4–**^ recorded in an OTTLE cell: (a) during the progressive decrease of the electrode potential from −0.40 to −2.0 V (vs Ag pseudoreference electrode) at a scan rate of 1 mV s^–1^; (b) before (black line) and after (red line) CV from–0.40 to −2.0 V (scan rate: 1 mV s^–1^). [N^*n*^Bu_4_][PF_6_] (0.1 mol dm^–3^) is the supporting electrolyte. The absorptions of the solvent and supporting electrolyte have been subtracted.

The spectra reported in Figure 11a were sequentially collected in the potential range −0.40 to −2.0 V (vs Ag pseudoreference electrode) at a scan rate of 1 mV s^–1^. The decrease of the applied potential produced a continuous and gradual shift to lower wavenumbers of both ν^t^_CO_ and ν^b^_CO_. The potential can be cycled between −0.40 and −1.5 V with no decomposition of the electrogenerated species, as demonstrated by the fact that the IR spectrum, recorded when the working electrode potential was returned to the initial value, is superimposable with that of the starting **1**^**4–**^. When the working electrode potential was further decreased to −2.0 V, the reduction of the cluster was accompanied by a relatively slow transformation into a species that does not quantitatively restore the spectrum of the starting tetraanion in the reverse oxidation back-scan (Figure 11b).

**Figure 12:**
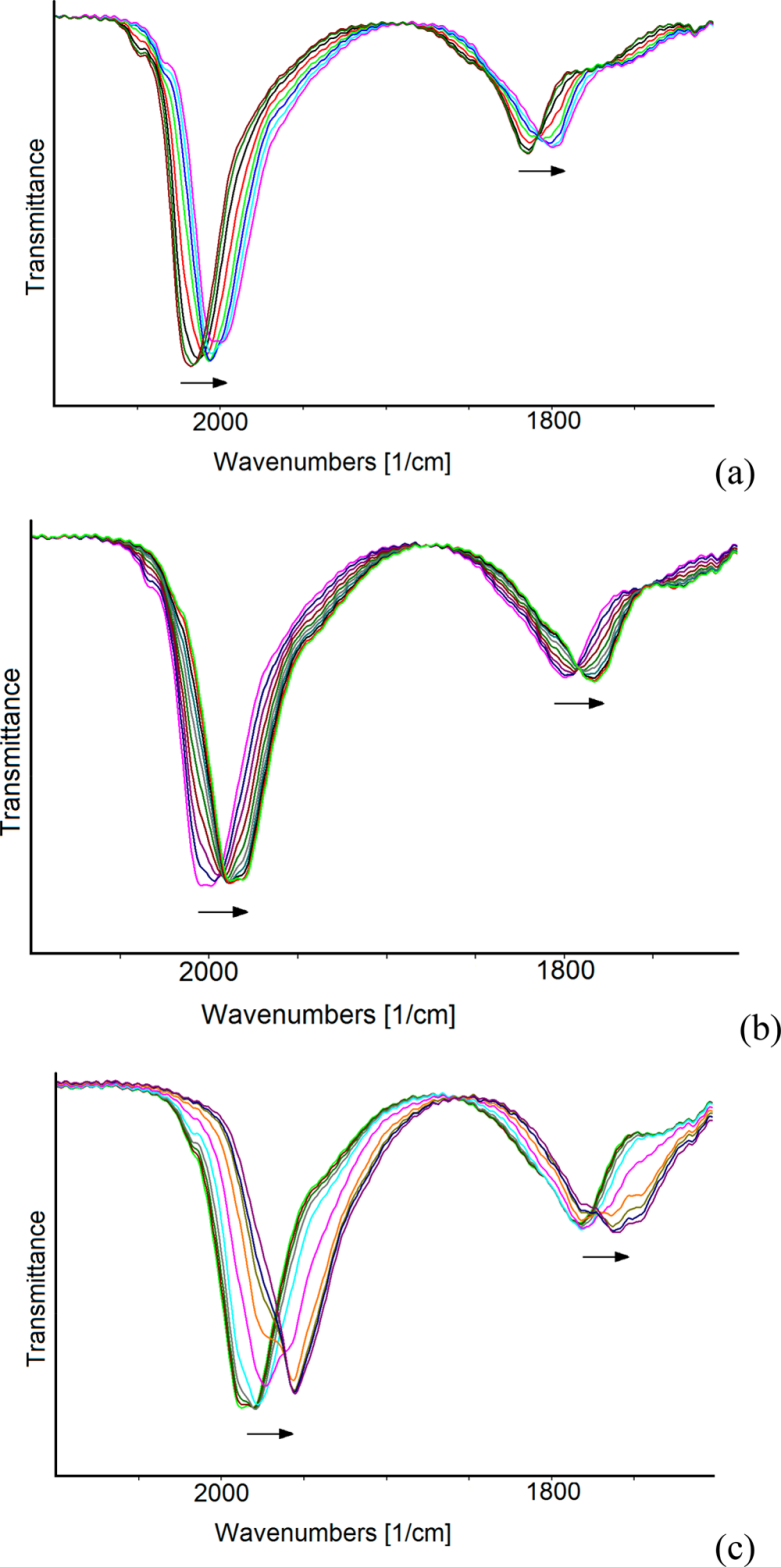
IR spectral changes of a CH_3_CN solution of **1**^**4–**^ recorded in an OTTLE cell during the progressive decrease of the potential from (a) – 0.40 to −0.90 V, (b) from −0.90 to −1.44 V, and (c) from −1.44 to −2.0 V (vs Ag pseudoreference electrode). [N^*n*^Bu_4_][PF_6_] (0.1 mol dm^–3^) is the supporting electrolyte. The absorptions of the solvent and supporting electrolyte have been subtracted.

The sequence of IR spectra of Figure 11a was analyzed and separated into three groups corresponding to three different redox steps (Figure 12), taking into account the profile of the related *i*/*E* curve and absorbance maxima in the bridging CO stretching zone. The first reduction occurs in the potential range from −0.40 to −0.90 V; the IR bands of **1**^**4–**^ shift at lower frequencies (from 2018, 1814, and 1777 cm^–1^ to 2004, 1999, 1803, and 1756 cm^–1^; Figure 12a), and fairly defined isosbestic points are observable in this spectra, indicating the relative stability of the electrogenerated species.

A second reduction process is evident from the spectra of Figure 12b. In this case, in the potential range from −0.90 to −1.44 V, the carbonyl absorptions downshift to 1988, 1981, 1783, and 1733 cm^–1^ with well-defined isosbestic points.

Finally, a third reduction process occurs between −1.44 and −2.0 V (Figure 12c). The progressive lowering of ν_CO_ bands to 1955, 1780, 1762, and 1748 cm^–1^ occurs without maintaining isosbestic points. Moreover, the bands broaden upon reduction, and the relative intensities of ν^t^_CO_ and ν^b^_CO_ vary, in agreement with the observation that an increasing negative charge on the cluster promotes the bridging coordination mode of the CO ligands.^63,64^

The ν^t^_CO_ and ν^b^_CO_ bands of the reversible stable redox states of the cluster are reported in Table 1. The charge of the electrogenerated species was assigned based on the change of the ν^t^_CO_ bands: a shift of 14–20 cm^–1^ indicates a one-electron step for high-nuclearity metal carbonyl clusters,^36,65−67^ whereas a shift of about 28 cm^–1^ is related to the bielectronic processes.^34,39^

**Table 1:** IR Frequencies (cm^–1^) of the Terminal (ν^t^_CO_) and Bridging (ν_CO_^b^) Carbonyl Groups for **1**^***n*–**^ in CH_3_CN as a Function of the Cluster Charge *n*^a^

cluster charge *n*	ν_CO_^t^	ν_CO_^b^
**–3**	2037	1832, 1797
**–4**	2018	1814, 1777
**–5**	2004, 1999	1803, 1756
**–6**	1988, 1981	1783, 1733
***–7***	*1975*	*1782*
**–8**	1955	1780, 1762, 1748

a The row in italics corresponds to the cluster charge deduced by spectral deconvolution.

In this regard, we can note that, in the potential range from −1.44 to −2.0 V (Figure 12c), the ν^t^_CO_ shift is about 30 cm^–1^, and the corresponding pattern of the spectra is complicated and without an isosbestic point. This suggests the presence of an intermediate transient negative state. Deconvolution analysis on two selected intermediate spectra of the reduction sequence reported in Figure 12c allowed determination of the two absorbance contributions at 1975 and 1955 cm^–1^ that give a good fitting (Figure S9). The combination ratio of the two bands changes according to the potential direction scan with a maximum area of 49% in favor of the higher-frequency band, which we tentatively assigned to the terminal CO groups of **1**^**7–**^.

**Figure 13:**
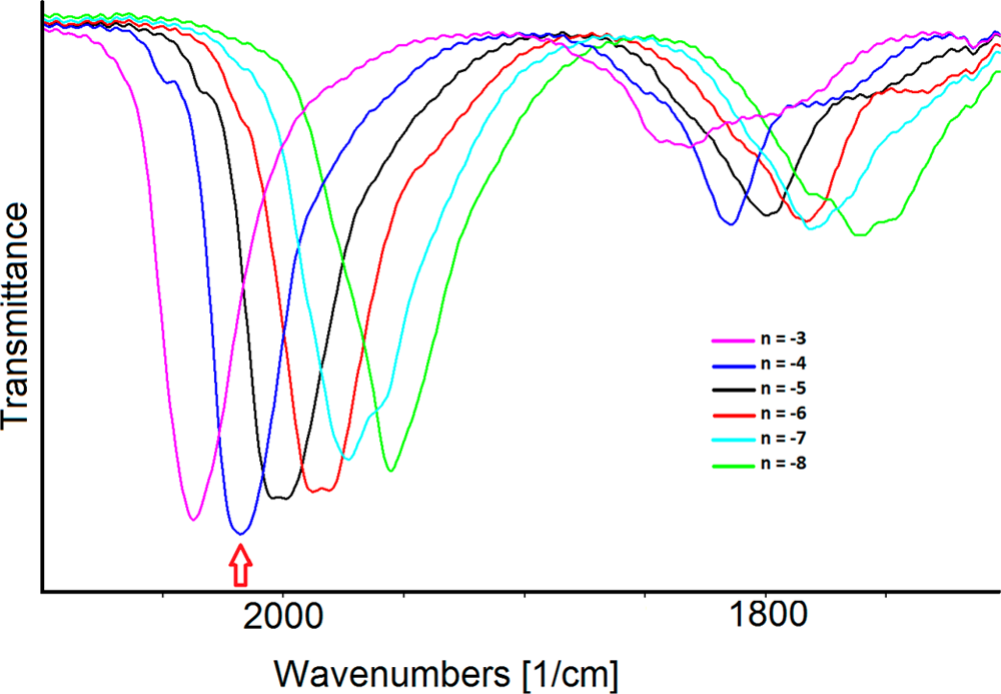
IR spectra of **1**^**4–**^ recorded in an OTTLE cell during a progressive decrease of the potential from +0.24 to −2.0 V (vs Ag pseudoreference electrode) in CH_3_CN containing 0.1 mol dm^–3^ [N^*n*^Bu_4_][PF_6_] is the supporting electrolyte. The absorptions of the solvent and supporting electrolyte have been subtracted. The red arrow indicates the initial spectrum.

Selected IR spectra assigned to the three long-lived redox states of **1**^***n*–**^ (4–, 5–, and 6−), together with those of two species (3– and 8−) with limited stability in the IR SEC time scale, are shown in Figure 13. We also gathered evidence of a cluster with charge 7–, which does not accumulate to be the predominant species in solution, and in Figure 13, we report the spectrum with the highest concentration obtained in our conditions.

**Figure 14:**
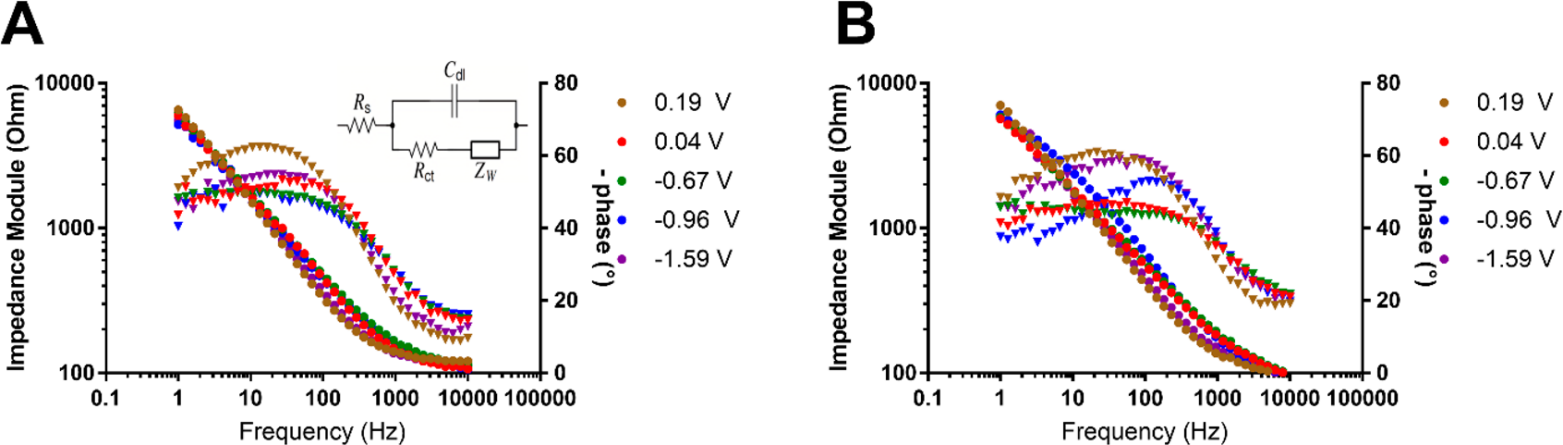
EIS spectra reported as Bode plots for the (A) GC and (B) Pt electrodes. The working dc potentials for each curve were 0.19 V (brown circles), 0.04 V (red circles), −0.67 V (green circles), −0.96 V (blue circles), and −1.59 V (purple circles).

EIS analysis was performed using as *E*_dc_ the *E*°′ calculated from the CV reported in Figure 9. Figure 14 shows the Bode plots of each spectrum recorded at +0.19, +0.04, −0.67, −0.96, and −1.59 V (vs Ag/AgCl, KCl sat.) for GC and Pt working electrodes.

As can be seen from the graph, the impedance module (circles) of the EIS spectra does not show any significant difference when the behaviors of **1**^**4**–^ are compared at the GC and Pt working electrodes. More information can be extracted from analysis of the phase (triangles), also reported in Figure 14. The processes at the GC electrode exhibit the same behavior, except for the peak corresponding to the second oxidation (+0.19 V), which shows a significant variation in the phase shift of the system at lower frequency (i.e., the shift related to the charge-transfer resistance of the equivalent circuit in the inset of Figure 14A), which we theorize is related to the chemical complications following the oxidation proven by the IR SEC experiment. The spectra recorded at the Pt electrode present a behavior similar to that observed at the GC working electrode, for all peaks except for the reduction processes at −0.96 and −1.59 V. While the process at −0.96 V did not show any abnormal behavior with IR SEC and CV analyses, the phase shift of the peak at −1.59 V can be related to the poorly defined peak in the CV and the unresolved **1**^**7**–^ redox state of the cluster shown by the IR SEC.

## 4 Conclusions

The new atomically precise Pt nanocluster **1**^**4**–^ has been obtained by a thermal method and fully characterized by a multitechnique approach involving IR and UV–visible spectroscopy, SC-XRD, dc SQUID magnetometry, CV, IR SEC, and EIS, as well as computational investigations. Its metal core displays a defective ccp structure, and local deformations occur in correspondence with this vacancy to fix the defect and lower the total energy. A similar behavior was previously observed for other molecular Pt nanoclusters, and, thus, it seems to be a general mechanism with possible involvement in all processes promoted by small metal nanoclusters.

The compound is perfectly diamagnetic, and its electronic spectrum shows continuous interband absorptions. The electrochemical behavior of **1**^**4**–^ indicates an incipient metalization of its metal core. Indeed, six oxidation states have been characterized by IR SEC, corresponding to five monoelectronic steps (one oxidation and four reductions). Thus, **1**^**4**–^ is multivalent and displays an electron-sink behavior, which leads to the **1**^***n***–^ (*n* = 3–8) series of isostructural nanoclusters. These oxidation states display different stabilities, and, in particular, **1**^**7**–^ seems to be very elusive. This is indicative of the fact that, within this size regime, the energetic levels of the cluster are close enough to allow reversible addition/removal of electrons but, at the same time, still sufficiently separated to clearly distinguish among the different oxidation states of **1**^***n***–^ (*n* = 3–8).

## Supporting Information Available

Cartesian coordinates of the computationally optimized structures (XYZ)
Supplementary IR-SEC figures and crystal data and collection details (PDF)

## Supplementary Material

ic2c00965_si_001.xyz

## References

[ref1] Jadzinsky P. D.; Calero G.; Ackerson C. A.; Bushnell D. A.; Kornberg R. D. Structure of a Thiol Monolayer-Protected Gold Nanoparticle at 1.1 Å Resolution. Science 2007, 318, 430–433. 10.1126/science.1148624. 17947577

[ref2] Jin R.; Zeng C.; Zhou M.; Chen Y. Atomically Precise Colloid Metal Nanoclusters and Nanoparticles: Fundamentals and Opportunities. Chem. Rev. 2016, 116, 10346–10413. 10.1021/acs.chemrev.5b00703. 27585252

[ref3] Chakraborty I.; Pradeep T. Atomically Precise Clusters of Noble Metals: Emerging Link between Atoms and Nanoparticles. Chem. Rev. 2017, 117, 8208–8271. 10.1021/acs.chemrev.6b00769. 28586213

[ref4] Cesari C.; Shon J.-H.; Zacchini S.; Berben L. A. Metal carbonyl clusters of groups 8–10: synthesis and catalysis. Chem. Soc. Rev. 2021, 50, 9503–9539. 10.1039/D1CS00161B. 34259674

[ref5] Li Y.; Zhou M.; Jin R. Programmable Metal Nanoclusters with Atomic Precision. Adv. Mater. 2021, 33, 2006591 10.1002/adma.202006591. 33984169

[ref6] Zhou M.; Du X.; Wang H.; Jin R. The Critical Number of Gold Atoms for a Metallic State Nanocluster: Resolving a Decades-Long Question. ACS Nano 2021, 15, 13980–13992. 10.1021/acsnano.1c04705. 34490772

[ref7] Jin R.; Li G.; Sharma S.; Li Y.; Du X. Toward Active-Site Tailoring in Heterogeneous Catalysis by Atomically Precise Metal Nanoclusters with Crystallographic Structures. Chem. Rev. 2021, 121, 567–648. 10.1021/acs.chemrev.0c00495. 32941029

[ref8] Kawawaki T.; Ebina A.; Hosokawa Y.; Ozaki S.; Suzuki D.; Hossain S.; Negishi Y. Thiolate-Protected Metal Nanoclusters: Recent Development in Synthesis, Understanding of Reaction, and Application in Energy and Environmental Field. Small 2021, 17, 2005328 10.1002/smll.202005328. 33522090

[ref9] Kawawaki T.; Kataoka Y.; Hirata M.; Iwamatsu Y.; Hossain S.; Negishi Y. Toward the creation of high-performance heterogeneous catalysts by controlled ligand desorption from atomically precise metal nanoclusters. Nanoscale Horiz. 2021, 6, 409–448. 10.1039/D1NH00046B. 33903861

[ref10] Kawawaki T.; Imai Y.; Suzuki D.; Kato S.; Kobayashi I.; Suzuki T.; Kaneko R.; Hossain S.; Negishi Y. Atomically Precise Alloy Nanoclusters. Chem. Eur. J. 2020, 26, 16150–16193. 10.1002/chem.202001877. 32453462

[ref11] Sun C.; Teo B. K.; Deng C.; Lin J.; Luo G.-G.; Tung C.-H.; Sun D. Hydrido-coinage-metal clusters: Rational design, synthetic protocols and structural characteristic. Coord. Chem. Rev. 2021, 427, 213576 10.1016/j.ccr.2020.213576.

[ref12] Du Y.; Sheng H.; Astruc D.; Zhu M. Atomically Precise Noble Metal Nanoclusters as Efficient Catalysts: A Bridge between Structure and Properties. Chem. Rev. 2020, 120, 526–622. 10.1021/acs.chemrev.8b00726. 30901198

[ref13] Femoni C.; Iapalucci M. C.; Longoni G.; Tiozzo C.; Zacchini S. An Organometallic Approach to Gold Nanoparticles: Synthesis and X-Ray Structure of CO-Protected Au_21_Fe_10_, Au_22_Fe_12_, Au_28_Fe_14_, and Au_34_Fe_14_ Clusters. Angew. Chem., Int. Ed. 2008, 47, 6666–6669. 10.1002/anie.200802267. 18651632

[ref14] Ciabatti I.; Femoni C.; Iapalucci M. C.; Ruggieri S.; Zacchini S. The role of gold in transition metal carbonyl clusters. Coord. Chem. Rev. 2018, 355, 27–38. 10.1016/j.ccr.2017.07.011.

[ref15] Biroju R. K.; Harrison P.; Theis W.; Rees N. V.; Sharma R.; Narayanan T. N.; Hahm M. G. Pt_147_ Nanoclusters Soft-Landed on WS_2_ Nanosheets for Catalysis and Energy Harvesting. ACS Appl. Nano Mater. 2021, 4, 13140–13148. 10.1021/acsanm.1c02683.

[ref16] Tsunoyama H.; Ohnuma A.; Takahashi K.; Velloth A.; Ehara M.; Ichikuni N.; Tabuchi M.; Nakajima A. Enhanced oxygen reduction activity of platinum subnanocluster catalysts through charge redistribution. Chem. Commun. 2019, 55, 12603–12606. 10.1039/C9CC06327G. 31556435

[ref17] Fracchia M.; Ghigna P.; Marelli M.; Scavini M.; Vertova A.; Rondinini S.; Della Pergola R.; Minguzzi A. Molecular cluster route for the facile synthesis of a stable and active Pt nanoparticle catalyst. New J. Chem. 2021, 45, 11292–11303. 10.1039/D1NJ00937K.

[ref18] Nair A. S.; Anoop A.; Ahuja R.; Pathak B. Role of atomicity in the oxygen reduction reaction activity of platinum sub nanometer clusters: A global optimization study. J. Comput. Chem. 2021, 42, 1944–1958. 10.1002/jcc.26725. 34309891

[ref19] Cheng Q.; Hu C.; Wang G.; Zou Z.; Yang H.; Dai L. Carbon-Defect-Driven Electroless Deposition of Pt Atomic Clusters for Highly Efficient Hydrogen Evolution. J. Am. Chem. Soc. 2020, 142, 5594–5601. 10.1021/jacs.9b11524. 32088958

[ref20] Imaoka T.; Akanuma Y.; Haruta N.; Tsuchiya S.; Ishihara K.; Okayasu T.; Chun W.-J.; Takahashi M.; Yamamoto K. Platinum clusters with precise numbers of atoms for preparative-scale catalysis. Nat. Commun. 2017, 8, 688 10.1038/s41467-017-00800-4. 28947792 PMC5613004

[ref21] Deraedt C.; Melaet G.; Ralston W. T.; Ye R.; Somorjai G. A. ″Platinum and Other Transition Metal Nanoclusters (Pd, Rh) Stabilized by PAMAM Dendrimers as Excellent Heterogenoeous Catalysts: Application to the Methylcyclopentane (MCP) Hydrogenative Isomerization. Nano Lett. 2017, 17, 1853–1862. 10.1021/acs.nanolett.6b05156. 28151681

[ref22] Tsunoyama H.; Yamano Y.; Zhang C.; Komori M.; Eguchi T.; Nakajima A. Size-Effect on Electrochemical Hydrogen Evolution Reaction by Single-Size Platinum Nanocluster Catalysts Immobilized on Strontium Titanate. Top. Catal. 2018, 61, 126–135. 10.1007/s11244-018-0884-7.

[ref23] Rodríguez-Kessler P. L.; Rodríguez-Domínguez A. R. Size and structure effects of Pt_N_ (N = 12–13) clusters for the oxygen reduction reaction: First-principles calculations. J. Chem. Phys. 2015, 143, 184312 10.1063/1.4935566. 26567667

[ref24] Imaoka T.; Kitazawa H.; Chun W.-J.; Omura S.; Albrecht K.; Yamamoto K. Magic Number Pt_13_ and Misshapen Pt_12_ Clusters: Which One is the Better Catalyst?. J. Am. Chem. Soc. 2013, 135, 13089–13095. 10.1021/ja405922m. 23902457

[ref25] Kratzl K.; Kratky T.; Günther S.; Tomanec O.; Zbořil R.; Michalička J.; Macak J. M.; Cokoja M.; Fischer R. A. Generation and Stabilization of Small Platinum Clusters Pt_12±x_ Inside a Metal-Organic Framework. J. Am. Chem. Soc. 2019, 141, 13962–13969. 10.1021/jacs.9b07083. 31398974

[ref26] Garlyyev B.; Kratzl K.; Rück M.; Michalička J.; Fichtner J.; Macak J. M.; Kratky T.; Günther S.; Cokoja M.; Bandarenka A. S.; Gagliardi A.; Fischer R. A. Optimizing the Size of Platinum Nanoparticles for Enhanced Mass Activity in the Electrochemical Oxygen Reduction Reaction. Angew. Chem., Int. Ed. 2019, 58, 9596–9600. 10.1002/anie.201904492. 31050857

[ref27] Ciabatti I.; Femoni C.; Iapalucci M. C.; Longoni G.; Zacchini S. Platinum Carbonyl Clusters Chemistry: Four Decades of Challenging Nanoscience. J. Clust. Sci. 2014, 25, 115–146. 10.1007/s10876-013-0639-3.

[ref28] Berti B.; Femoni C.; Iapalucci M. C.; Ruggieri S.; Zacchini S. Functionalization, Modification, and Transformation of Platinum Chini Clusters. Eur. J. Inorg. Chem. 2018, 2018, 3285–3296. 10.1002/ejic.201800526.

[ref29] Berti B.; Bortoluzzi M.; Ceriotti A.; Cesari C.; Femoni C.; Carmela Iapalucci M.; Zacchini S. Further insights into platinum carbonyl Chini clusters. Inorg. Chim. Acta 2020, 512, 119904 10.1016/j.ica.2020.119904.

[ref30] Bortoluzzi M.; Cesari C.; Ciabatti I.; Femoni C.; Iapalucci M. C.; Zacchini S. Reactions of Platinum Carbonyl Chini Clusters with Ag(NHC)Cl Complexes: Formation of Acid-Base Lewis Adducts and Heteroleptic Clusters. Inorg. Chem. 2017, 56, 6532–6544. 10.1021/acs.inorgchem.7b00665. 28489358

[ref31] Femoni C.; Iapalucci M. C.; Longoni G.; Lovato T.; Stagni S.; Zacchini S. Self-Assembly of [Pt_3n_(CO)_6n_]^2–^ (n = 4–8) Carbonyl Clusters; from Molecules to Conducting Molecular Metal Wires. Inorg. Chem. 2010, 49, 5992–6004. 10.1021/ic100547j. 20536247

[ref32] Berti B.; Cesari C.; Conte F.; Ciabatti I.; Femoni C.; Iapalucci M. C.; Vacca F.; Zacchini S. Synthesis of [Pt_12_(CO)_20_(dppm)_2_]^2–^ and [Pt_18_(CO)_30_(dppm)_3_]^2–^ Heteroleptic Chini-Type Platinum Clusters by the Oxidative Oligomerization of [Pt_6_(CO)_10_(dppm)]^2–^. Inorg. Chem. 2018, 57, 7578–7590. 10.1021/acs.inorgchem.8b00447. 29889503

[ref33] Barnett B. R.; Rheingold A. I.; Figueroa J. S. Monomeric Chini-type triplatinum clusters featuring dianionic and radical-anionic π*-systems. Angew. Chem., Int. Ed. 2016, 55, 9253–9258. 10.1002/anie.201604903. 27346691

[ref34] Cattabriga E.; Ciabatti I.; Femoni C.; Funaioli T.; Iapalucci M. C.; Zacchini S. Syntheses, Structures, and Electrochemistry of the Defective *ccp* [Pt_33_(CO)_38_]^2–^ and the *bcc* [Pt_40_(CO)_40_]^6–^ Molecular Nanoclusters. Inorg. Chem. 2016, 55, 6068–6079. 10.1021/acs.inorgchem.6b00607. 27281686

[ref35] Cattabriga E.; Ciabatti I.; Femoni C.; Iapalucci M. C.; Longoni G.; Zacchini S. Globular molecular platinum carbonyl nanoclusters: Synthesis and molecular structures of the [Pt_26_(CO)_32_]^−^ and [Pt_14+x_(CO)_18+x_]^4–^ anions and their comparison to related platinum ″browns″. Inorg. Chim. Acta 2018, 470, 238–249. 10.1016/j.ica.2017.04.045.

[ref36] Fedi S.; Zanello P.; Laschi F.; Ceriotti A.; El Afefey S. A joint electrochemical/spectroelectrochemical inspection (and re-inspection) of high-nuclearity platinum carbonyl clusters. J. Solid State Electrochem. 2009, 13, 1497–1504. 10.1007/s10008-009-0880-8.

[ref37] Ceriotti A.; Masciocchi N.; Macchi P.; Longoni G. [Pt_19_(CO)_21_(NO)]^3–^ and [Pt_38_(CO)_44_]^2–^: Nitrosyl bending through intramolecular electron transfer as an intermediate step in the nucleation process from polydecker to ccp platinum carbonyl clusters. Angew. Chem., Int. Ed. 1999, 38, 3724–3727. 10.1002/(SICI)1521-3773(19991216)38:24<3724::AID-ANIE3724>3.0.CO;2-S.

[ref38] Lewis G. J.; Roth J. D.; Montag R. A.; Safford L. K.; Gao X.; Chang S.-C.; Dahl L. F.; Weaver M. J. Electroactive Metal Clusters as Models of Electrode Surfaces: Vibrational Spectroelectrochemistry of Seven Redox Forms of [Pt_24_(CO)_30_]^n^ (n = 0 to – 6) and Comparison with Potential-Dependent Spectra of CO Chemisorbed on Platinum. J. Am. Chem. Soc. 1990, 112, 2831–2832. 10.1021/ja00163a071.

[ref39] Roth J. D.; Lewis G. J.; Safford L. K.; Jiang X.; Dahl L. F.; Weaver M. J. Exploration of Ionizable Metal Cluster-Electrode Surface Analogy: Infrared Spectroelectrochemistry of [Pt_24_(CO)_30_]^n^, [Pt_26_(CO)_32_]^n^, and [Pt_38_(CO)_44_]^n^ (n = 0 to – 10) and Comparison with Potential-Dependent Spectra of CO Adlayers of Platinum Surfaces. J. Am. Chem. Soc. 1992, 114, 6159–6169. 10.1021/ja00041a038.

[ref40] Washecheck D. M.; Wucherer E. J.; Dahl L. F.; Ceriotti A.; Longoni G.; Manassero M.; Sansoni M.; Chini P. Synthesis, Structure, and Stereochemical Implication of the [Pt_19_(CO)_12_(m_2_-CO)_10_]^4–^ Tetraanion: A Bicapped Triple-Decker All-Metal Sandwich of Idealized Fivefold (D_5h_) Geometry. J. Am. Chem. Soc. 1979, 101, 6110–6112. 10.1021/ja00514a039.

[ref41] Jin R.; Higaki T. Open questions on the transition between nanoscale and bulk properties of metals. Commun. Chem. 2021, 4, 28 10.1038/s42004-021-00466-6. PMC9814084 36697528

[ref42] Zacchini S. Using Metal Carbonyl Clusters To Develop a Molecular Approach towards Metal Nanoparticles. Eur. J. Inorg. Chem. 2011, 2011, 4125–4145. 10.1002/ejic.201100462.

[ref43] Cesari C.; Funaioli T.; Berti B.; Femoni C.; Iapalucci M. C.; Vivaldi F. M.; Zacchini S. Atomically Precise Ni-Pd Alloy Carbonyl Nanoclusters: Synthesis, Total Structure, Electrochemistry, Spectroelectrochemistry, and Electrochemical Impedance Spectroscopy. Inorg. Chem. 2021, 60, 16713–16725. 10.1021/acs.inorgchem.1c02582. 34672566 PMC8564757

[ref44] Cesari C.; Femoni C.; Funaioli T.; Iapalucci M. C.; Rivalta I.; Ruggieri S.; Zacchini S. Heterometallic rhodium clusters as electron reservoirs: Chemical, electrochemical, and theoretical studies of the centered-icosahedral [Rh_12_E(CO)_27_]^n–^ atomically precise carbonyl compounds. J. Chem. Phys. 2021, 155, 104301 10.1063/5.0061764. 34525822

[ref45] Longoni G.; Chini P. Synthesis and chemical characterization of platinum carbonyl dianions [Pt_3_(CO)_6_]_n_^2–^ (n = ∼ 10, 6, 5, 4, 3, 2, 1). A new series of inorganic oligomers. J. Am. Chem. Soc. 1976, 98, 7225–7231. 10.1021/ja00439a020.

[ref46] Keller E. SCHAKAL99; University of Freiburg: Freiburg, Germany, 1999.

[ref47] Sheldrick G. M. SADABS-2008/1: Bruker AXS Area Detector Scaling and Absorption Correction; Bruker AXS: Madison, WI, 2008.

[ref48] Sheldrick G. M. Crystal structure refinement with SHELXL. Acta Crystallogr., Sect. C: Struct. Chem. 2015, 71, 3–8. 10.1107/S2053229614024218. 25567568 PMC4294323

[ref49] Spek A. L. Single-crystal structure validation with the program. PLATON. J. Appl. Crystallogr. 2003, 36, 7–13. 10.1107/S0021889802022112.

[ref50] Spek A. L. Structure validation in chemical crystallography. Acta. Crystallogr., Sect. D: Biol. Crystallogr. 2009, D65, 148–155. 10.1107/S090744490804362X. PMC2631630 19171970

[ref51] Bannwarth C.; Ehlert S.; Grimme S. GFN2-xTB∏An Accurate and Broadly Parametrized Self-Consistent Tight-Binding Quantum Chemical Method with Multipole Electrostatics and Density-Dependent Dispersion Contributions. J. Chem. Theory Comput. 2019, 15, 1652–1671. 10.1021/acs.jctc.8b01176. 30741547

[ref52] Ehlert S.; Stahn M.; Spicher S.; Grimme S. Robust and Efficient Implicit Solvation Model for Fast Semiempirical Methods. J. Chem. Theory Comput. 2021, 17, 4250–4261. 10.1021/acs.jctc.1c00471. 34185531

[ref53] aGrimme S.; Brandenburg J. G.; Bannwarth C.; Hansen A. Consistent structures and interactions by density functional theory with small atomic orbital basis sets. J. Chem. Phys. 2015, 143, 054107 10.1063/1.4927476. 26254642

[ref54] Bannwarth C.; Caldeweyher E.; Ehlert S.; Hansen A.; Pracht P.; Seibert J.; Spicher S.; Grimme S. Extended tight-binding quantum chemistry methods. WIREs Comput. Mol. Sci. 2020, 11, e01493 10.1002/wcms.1493.

[ref55] Neese F. Software update: The ORCA program system—Version 5.0. WIREs Comput. Mol. Sci. 2022, e1616 10.1002/wcms.1606.

[ref56] Lu T.; Chen F. Multiwfn: A multifunctional wavefunction analyzer. J. Comput. Chem. 2012, 33, 580–592. 10.1002/jcc.22885. 22162017

[ref57] Allouche A.-R. Gabedit - A graphical user interface for computational chemistry softwares. J. Comput. Chem. 2011, 32, 174–182. 10.1002/jcc.21600. 20607691

[ref58] Bianchi R.; Gervasio G.; Marabello D. Experimental Electron Density Analysis of Mn_2_(CO)_10_: Metal-Metal and Metal-Ligand Bond Characterization. Inorg. Chem. 2000, 39, 2360–2366. 10.1021/ic991316e. 12526497

[ref59] Lepetit C.; Fau P.; Fajerwerg K.; Kahn M. L.; Silvi B. Topological analysis of the metal-metal bond: A tutorial review. Coord. Chem. Rev. 2017, 345, 150–162. 10.1016/j.ccr.2017.04.009.

[ref60] Hirshfeld F. L. Bonded-atom fragments for describing molecular charge densities. Theor. Chim. Acta 1977, 44, 129–138. 10.1007/BF00549096.

[ref61] Kawano M.; Bacon J. W.; Campana C. F.; Winger B. E.; Dudek J. D.; Sirchio S. A.; Scruggs S. L.; Geiser U.; Dahl L. F. High-Nuclearity Close-Packed Palladium-Nickel Carbonyl Phosphine Clusters: Heteropalladium [Pd_16_Ni_4_(CO)_22_(PPh_3_)_4_]^2–^ and [Pd_33_Ni_9_(CO)_41_(PPh_3_)_6_]^4–^ Containing Pseudo-*T*_*d*_ ccp Pd_14_Ni_4_ and Pseudo-*D*_*3h*_ hcp Pd_33_Ni_9_ Cores. Inorg. Chem. 2001, 40, 2554–2569. 10.1021/ic000979p. 11350234

[ref62] Krejčik M.; Daněk M.; Hartl F. Simple construction of an infrared optically transparent thin-layer electrochemical cell: Applications to the redox reactions of ferrocene, Mn_2_(CO)_10_ and Mn(CO)_3_(3,5.di-t-butyl-catecholate)^−^. J. Electroanal. Chem. 1991, 317, 179–187. 10.1016/0022-0728(91)85012-E.

[ref63] Ragaini F.; Song J.-S.; Ramage D. L.; Geoffroy G. L.; Yap G. A. P.; Rheingold A. L. Radical Processes in the Reduction of Nitrobenzene Promoted by Iron Carbonyl Clusters. X-ray Crystal Structures of [Fe_3_(CO)_9_(μ_3_-NPh)]^2–^, [HFe_3_(CO)_9_(μ_3_-NPh)]^−^, and the Radical Anion [Fe_3_(CO)_11_]^−^. Organometallics 1995, 14, 387–400. 10.1021/om00001a054.

[ref64] Cesari C.; Bortoluzzi M.; Femoni C.; Iapalucci M. C.; Zacchini S. One-pot atmospheric pressure synthesis of [H_3_Ru_4_(CO)_12_]^−^. Dalton Trans. 2021, 50, 9610–9622. 10.1039/D1DT01517F. 34160508

[ref65] Bortoluzzi M.; Ciabatti I.; Femoni C.; Funaioli T.; Hayatifar M.; Iapalucci M. C.; Longoni G.; Zacchini S. Homoleptic and heteroleptic Au(I) complexes containing the new [Co_5_C(CO)_12_]^−^ cluster as ligand. Dalton Trans. 2014, 43, 9633–9646. 10.1039/c4dt00854e. 24832236

[ref66] Ciabatti I.; Fabrizi de Biani F.; Femoni C.; Iapalucci M. C.; Longoni G.; Zacchini S. Metal Segregation in Bimetallic Co-Pd Carbide Carbonyl Clusters: Synthesis, Structure, Reactivity and Electrochemistry of [H_6-n_Co_20_Pd_16_C_4_(CO)_48_]^n–^ (n = 3–6). ChemPlusChem. 2013, 78, 1456–1465. 10.1002/cplu.201300268. 31986665

[ref67] Berti B.; Cesari C.; Femoni C.; Funaioli T.; Iapalucci M. C.; Zacchini S. Redox active Ni-Pd carbonyl alloy nanoclusters: syntheses, molecular structures and electrochemistry of [Ni_22-x_Pd_20+x_(CO)_48_]^6–^ (x = 0.62), [Ni_29-x_Pd_6+x_(CO)_42_]^6–^ (x = 0.09) and [Ni_29+x_Pd_6–x_(CO)_42_]^6–^ (x = 0.27). Dalton Trans. 2020, 49, 5513–5522. 10.1039/D0DT00337A. 32267267

