# Supplementary material for: Atomically Precise Platinum Carbonyl Nanoclusters: Synthesis, Total Structure, and Electrochemical Investigation of [Pt27(CO)31]4– Displaying a Defective Structure
Source: Inorg Chem. 2022 Aug 3;61(32):12534–44. doi: 10.1021/acs.inorgchem.2c00965 (PMC9387524; doi:10.1021/acs.inorgchem.2c00965)
Supplement: Supplementary file 2 — ic2c00965_si_002.pdf [file ic2c00965_si_002.pdf]

Supporting Information for

Atomically Precise Platinum Carbonyl Nanoclusters: Synthesis, Total Structure and Electrochemical Investigation of $[Pt_{27}(CO)_{31}]^{4-}$ Displaying a Defective Structure

Cristiana Cesari,[a] Beatrice Berti,[a] Tiziana Funaioli,[b] Cristina Femoni,[a] Maria Carmela Iapalucci,[a] Daniele Pontiroli,[c] Giacomo Magnani,[c] Mauro Riccò,[c] Marco Bortoluzzi,[d] Federico Maria Vivaldi [b] and Stefano Zacchini[a]*

[a] Dipartimento di Chimica Industriale "Toso Montanari", Università di Bologna, Viale Risorgimento 4 - 40136 Bologna, Italy. E-mail: stefano.zacchini@unibo.it

[b] Dipartimento di Chimica e Chimica Industriale, Università di Pisa, Via G. Moruzzi 13, 56124, Pisa

[c] Dipartimento di Scienze Matematiche, Fisiche e Informatiche & INSTM, Università degli Studi di Parma, Viale delle Scienze 7/a – 43124 Parma, Italy

[d] Dipartimento di Scienze Molecolari e Nanosistemi, Ca' Foscari University of Venice, Via Torino 155 – 30175 Mestre (Ve), Italy

	Page/s
X-Ray crystallographic data and details	S2
Supplementary DFT figures and Tables	S3-S9
Supplementary IR-SEC figures	S10-S12

Table S1. Crystal data and experimental details for $[PPh_4]_4[Pt_{27}(CO)_{31}]\cdot CH_3COCH_3\cdot solv$ and $[PPh_4][Pt_{26}(CO)_{32}]$.

	**$[PPh_4]_4[Pt_{27}(CO)_{31}]\cdot CH_3COCH_3\cdot solv$**	**$[PPh_4][Pt_{26}(CO)_{32}]$**
Formula	$C_{130}H_{86}O_{32}P_4Pt_{27}$	$C_{56}H_{20}O_{32}PPt_{26}$
Fw	7551.29	6308.03
T, K	100(2)	100(2)
λ, Å	0.71073	0.71073
Crystal system	Monoclinic	Monoclinic
Space Group	$P2_1/c$	$P2_1/n$
a, Å	25.578(2)	14.1272(10)
b, Å	16.2259(15)	33.871(2)
c, Å	34.800(3)	17.1790(12)
α, °	90	90
β, °	92.412(3)	95.836(2)
γ, °	90	90
Cell Volume, $Å^3$	14430(2)	8177.6(10)
Z	4	4
D_c, g cm^{-3}	3.476	5.124
μ, mm^{-1}	26.163	44.360
F(000)	13152	10620
Crystal size, mm	0.16×0.13×0.11	0.19×0.16×0.14
θ limits, °	1.487–23.999	1.569–25.050
Index ranges	$-29\leq h \leq 29$<br>$-18 \leq k \leq 18$<br>$-39\leq l \leq 39$	$-16\leq h \leq 16$<br>$-40 \leq k \leq 40$<br>$-20\leq l \leq 20$
Reflections collected	212478	145765
Independent reflections	22052 [R_{int} = 0.1779]	14381 [R_{int} = 0.0915]
Completeness to θ max	97.3%	99.2%
Data / restraints / parameters	22052 / 1239 / 1546	14381 / 1007 / 1114
Goodness on fit on F^2	1.159	1.164
R_1 ($I > 2\sigma(I)$)	0.1399	0.0853
wR_2 (all data)	0.3271	0.2049
Largest diff. peak and hole, e $Å^{-3}$	9.503 / –3.313	7.286 / –4.078

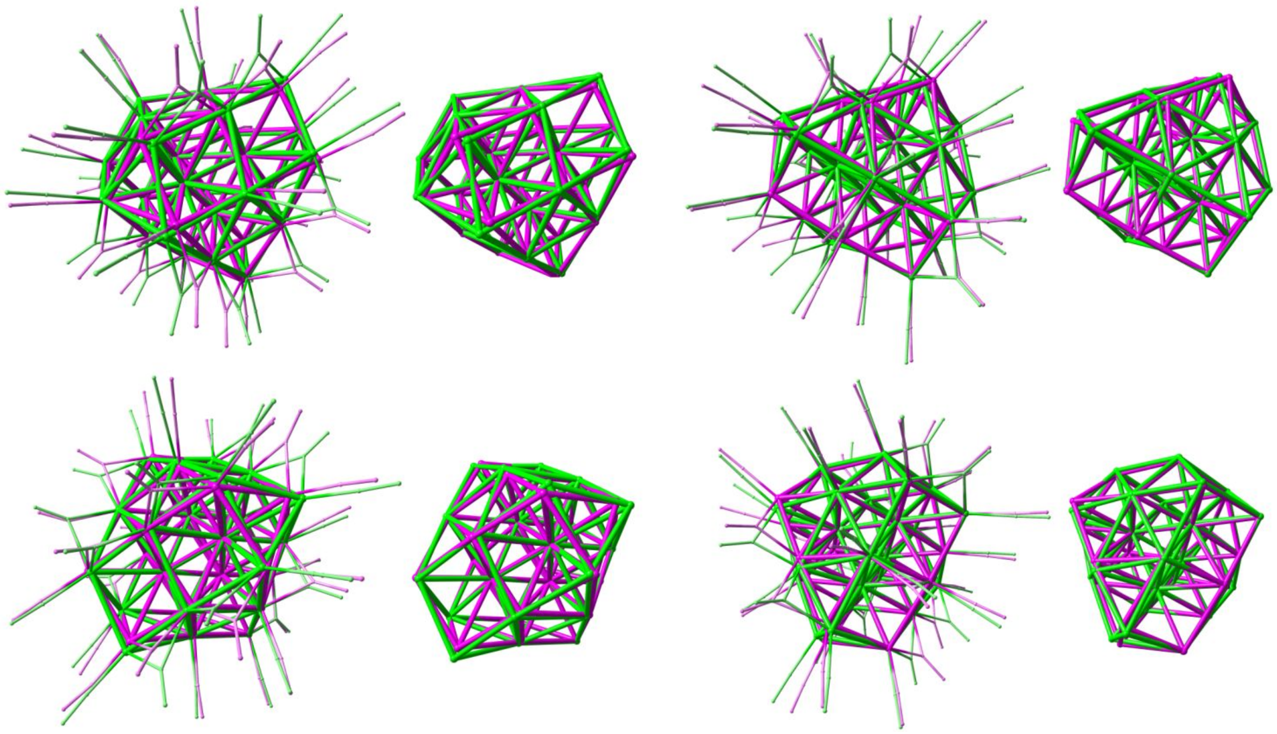

Figure S1. Different views of the best overlap between X-ray (purple) and PBEh-3c (green) structures of **1**$^{4-}$, with and without carbonyl ligands.

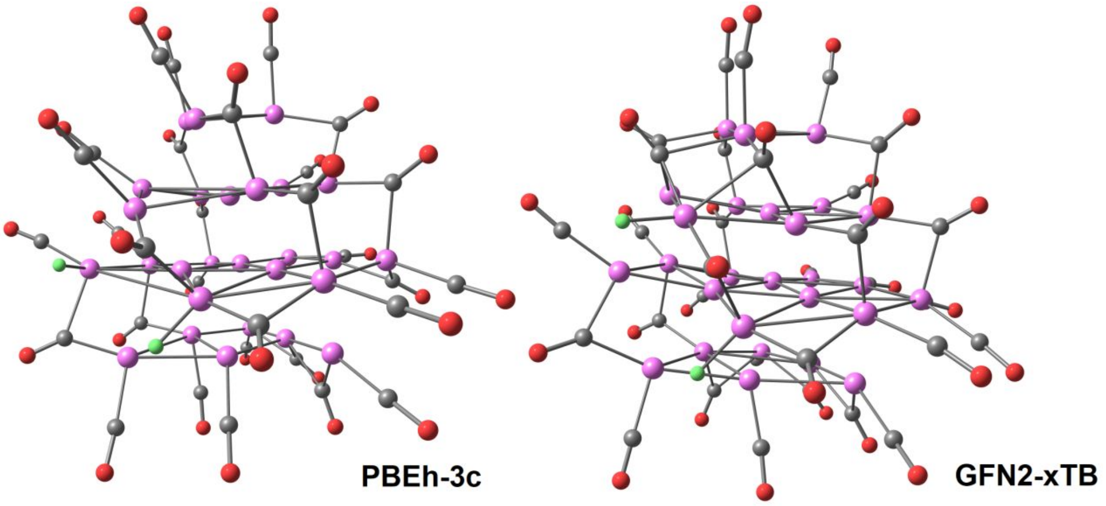

Figure S2. Computationally optimized structures of $[Pt_{27}H_2(CO)_{31}]^{4-}$ (purple, Pt; red, O; grey, C; green, H). The Pt-Pt interactions among the layers were not drawn for clarity. RMSD calculated for $\{Pt_{27}C_{31}O_{31}\}$ with respect to the experimental structure of **1**$^{4-}$: 0.611 Å (PBEh-3c), 0.788 Å (GFN2-xTB). RMSD calculated for $\{Pt_{27}\}$ with respect to the experimental structure of **1**$^{4-}$: 0.260 Å (PBEh-3c), 0.341 Å (GFN2-xTB).

Table S2. Selected data for the Pt-Pt (3,-1) b.c.p. in **1**$^{4-}$ (PBEh-3c optimized structure). ρ, e Å^{-3}; V, hartree Å^{-3}; E, hartree Å^{-3}; $\nabla^2\rho$, e Å^{-5}. Please refer to the main text for the numbering.

**b.c.p.**	**ρ**	**V**	**E**	**∇²ρ**	**b.c.p.**	**ρ**	**V**	**E**	**∇²ρ**
1	0.243	-0.216	-0.040	1.976	39	0.425	-0.391	-0.115	2.289
2	0.324	-0.317	-0.067	2.506	40	0.351	-0.324	-0.088	2.241
3	0.263	-0.216	-0.054	1.542	41	0.405	-0.398	-0.101	2.771
4	0.223	-0.196	-0.034	1.735	42	0.418	-0.378	-0.115	2.121
5	0.351	-0.047	-0.081	2.169	43	0.351	-0.324	-0.081	2.241
6	0.283	-0.317	-0.061	1.470	44	0.466	-0.493	-0.128	3.374
7	0.324	-0.310	-0.067	2.410	45	0.263	-0.216	-0.047	1.735
8	0.418	-0.391	-0.115	2.362	46	0.270	-0.236	-0.054	1.783
9	0.358	-0.364	-0.081	2.940	47	0.236	-0.209	-0.040	1.856
10	0.155	-0.115	-0.013	1.205	48	0.337	-0.290	-0.081	1.952
11	0.223	-0.029	-0.034	1.832	49	0.378	-0.351	-0.094	2.265
12	0.385	-0.196	-0.094	2.892	50	0.378	-0.358	-0.094	2.362
13	0.310	-0.263	-0.074	1.735	51	0.277	-0.209	-0.061	1.301
14	0.439	-0.432	-0.121	2.771	52	0.250	-0.216	-0.040	1.832
15	0.331	-0.317	-0.067	2.458	53	0.283	-0.263	-0.061	2.097
16	0.283	-0.263	-0.054	2.193	54	0.324	-0.283	-0.074	1.952
17	0.439	-0.425	-0.121	2.675	55	0.256	-0.216	-0.047	1.783
18	0.256	-0.202	-0.047	1.422	56	0.331	-0.317	-0.067	2.554
19	0.310	-0.304	-0.061	2.530	57	0.229	-0.202	-0.034	1.807
20	0.216	-0.182	-0.027	1.783	58	0.243	-0.209	-0.040	1.711
21	0.324	-0.283	-0.074	2.204	59	0.472	-0.472	-0.135	2.844
22	0.405	-0.364	-0.108	2.193	60	0.310	-0.283	-0.067	2.072
23	0.256	-0.229	-0.040	2.097	61	0.425	-0.391	-0.115	2.241
24	0.277	-0.229	-0.054	1.687	62	0.358	-0.351	-0.081	2.651
25	0.432	-0.452	-0.115	3.229	63	0.324	-0.283	-0.074	1.928
26	0.385	-0.371	-0.094	2.627	64	0.256	-0.236	-0.040	2.145
27	0.324	-0.304	-0.074	2.265	65	0.250	-0.189	-0.047	1.325
28	0.317	-0.283	-0.067	2.121	66	0.324	-0.290	-0.074	2.169
29	0.236	-0.189	-0.040	1.494	67	0.445	-0.412	-0.115	2.603
30	0.385	-0.391	-0.094	2.964	68	0.223	-0.189	-0.034	1.807
31	0.270	-0.243	-0.054	1.928	69	0.324	-0.277	-0.074	1.904
32	0.223	-0.189	-0.034	1.687	70	0.351	-0.331	-0.081	2.458
33	0.378	-0.364	-0.094	2.554	71	0.263	-0.223	-0.054	1.711
34	0.283	-0.256	-0.054	2.097	72	0.304	-0.263	-0.067	1.880
35	0.256	-0.263	-0.047	1.928	73	0.304	-0.277	-0.061	2.193
36	0.202	-0.162	-0.034	1.422	74	0.223	-0.196	-0.034	1.759
37	0.256	-0.223	-0.047	1.928	75	0.310	-0.263	-0.074	1.735
38	0.472	-0.459	-0.135	2.675	76	0.223	-0.189	-0.034	1.735

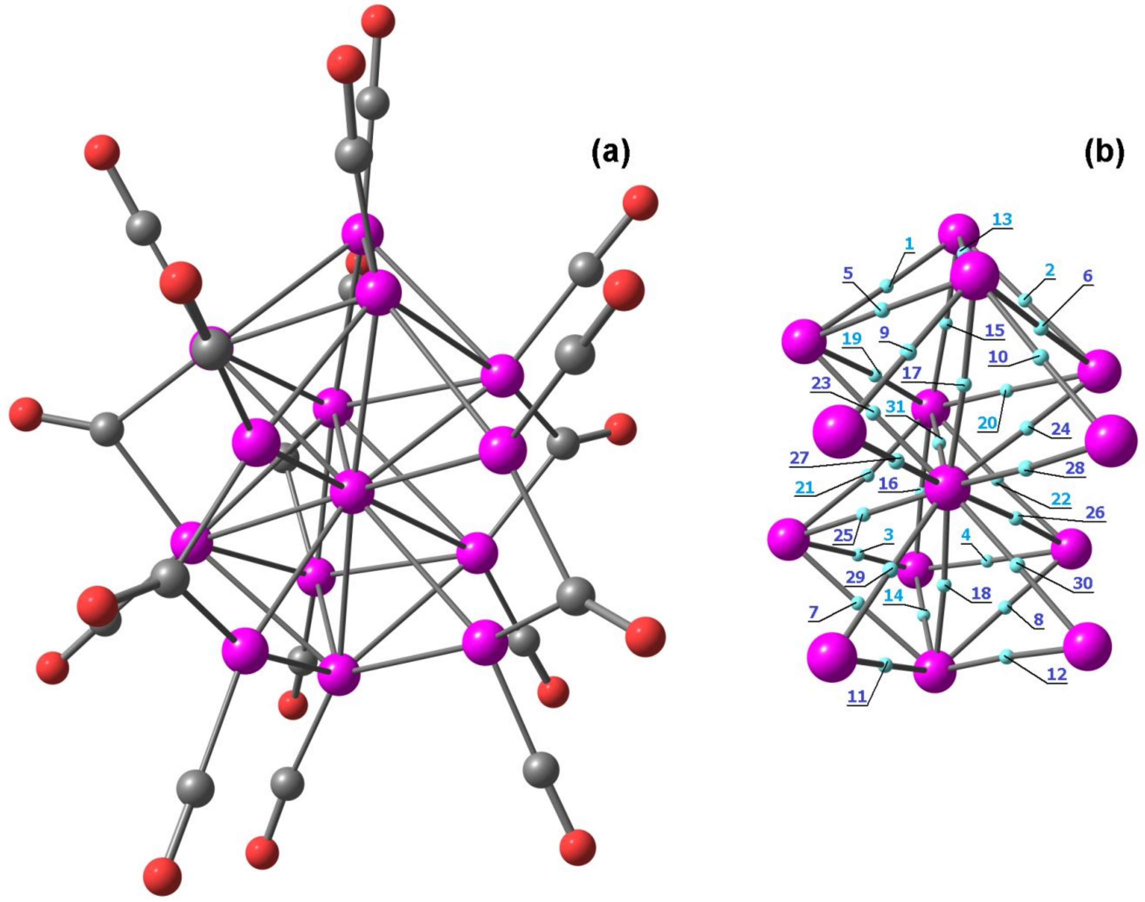

Figure S3. (a) DFT-optimized structure of $[Pt_{14}(CO)_{18}]^{4-}$ (PBEh-3c calculations). (b) $\{Pt_{14}\}$ core with Pt-Pt (3,-1) b.c.p. in cyan. Purple, Pt; red, O; grey, C. The b.c.p. numbers are colored accordingly to the ρ values: < 0.300 e $Å^{-3}$, light blue; > 0.300 e $Å^{-3}$ a.u., dark blue.

Table S3. Selected data for the Pt-Pt (3,-1) b.c.p. in $[Pt_{14}(CO)_{18}]^{4-}$ (PBEh-3c optimized structure). ρ, e $Å^{-3}$; V, hartree $Å^{-3}$; E, hartree $Å^{-3}$; $\nabla^2\rho$, e $Å^{-5}$. Please refer to the Figure S3 for the numbering.

b.c.p.	ρ	V	E	$\nabla^2\rho$	b.c.p.	ρ	V	E	$\nabla^2\rho$
1	0.228	-0.198	-0.037	1.778	17	0.384	-0.328	-0.106	1.664
2	0.252	-0.228	-0.043	2.036	18	0.402	-0.353	-0.112	1.851
3	0.218	-0.186	-0.034	1.695	19	0.211	-0.177	-0.030	1.653
4	0.251	-0.227	-0.043	2.014	20	0.207	-0.157	-0.033	1.307
5	0.379	-0.375	-0.089	5.479	21	0.193	-0.144	-0.028	1.268
6	0.381	-0.355	-0.098	2.264	22	0.214	-0.179	-0.033	1.619
7	0.354	-0.341	-0.080	2.589	23	0.408	-0.364	-0.109	2.085
8	0.389	-0.371	-0.099	2.464	24	0.398	-0.371	-0.100	2.438
9	0.351	-0.324	-0.082	2.298	25	0.426	-0.399	-0.115	2.423
10	0.390	-0.374	-0.099	2.520	26	0.343	-0.291	-0.081	1.838
11	0.395	-0.387	-0.096	2.784	27	0.444	-0.423	-0.123	2.537
12	0.379	-0.351	-0.097	2.258	28	0.365	-0.317	-0.091	1.943
13	0.257	-0.216	-0.046	1.785	29	0.396	-0.348	-0.105	1.978
14	0.259	-0.219	-0.046	1.818	30	0.408	-0.374	-0.107	2.291
15	0.408	-0.378	-0.094	2.708	31	0.228	-0.172	-0.038	1.362
16	0.410	-0.383	-0.095	2.747					

Table S4. Computed stretching frequencies related to the carbonyl ligands in **1**$^{4-}$.

GFN2-xTB		ALPB/GNF2-xTB	
Wavenumber (cm^{-1})	IR intensity (km mol^{-1})	Wavenumber (cm^{-1})	IR intensity (km mol^{-1})
1818.15	560.28	1730.10	30.14
1819.20	154.08	1791.65	135.27
1823.73	507.71	1800.48	166.22
1825.69	701.63	1806.65	82.86
1832.04	198.09	1810.46	271.06
1836.72	550.84	1815.15	46.44
1837.68	837.34	1816.50	66.93
1841.36	2484.34	1820.80	109.39
1864.93	51.93	1829.83	53.87
1868.89	417.24	1831.36	100.81
1871.20	296.81	1833.93	678.48
1880.55	2305.63	1845.11	336.35
1887.89	4041.51	1854.09	436.49
1892.02	3070.76	1858.35	758.61
1909.13	3084.93	1864.87	730.73
1941.94	41.51	1875.65	654.16
1950.17	320.09	1900.27	725.80
1956.34	145.90	1936.79	186.94
1964.79	179.86	1951.18	416.74
1965.19	965.55	1954.16	135.84
1965.52	789.07	1956.19	42.63
1969.58	1289.01	1957.79	24.34
1971.83	85.31	1965.39	269.66
1975.55	506.26	1972.31	34.14
1985.57	1294.04	1973.29	137.50
1993.97	1776.63	1978.20	205.45
2009.04	2574.77	1981.98	622.61
2020.88	6405.21	2042.28	7190.94
2030.24	13081.09	2055.07	5863.62
2035.66	17830.17	2065.40	4120.40
2071.67	409.68	2078.40	444.72

Table S5. Computed stretching frequencies related to the carbonyl ligands in $\mathbf{1}^{3-}$

**GFN2-xTB**		**ALPB/GNF2-xTB**	
Wavenumber (cm^{-1})	IR intensity (km mol^{-1})	Wavenumber (cm^{-1})	IR intensity (km mol^{-1})
1771.36	1537.90	1744.82	42.85
1843.41	127.09	1815.65	46.59
1847.31	101.12	1820.70	122.78
1850.77	356.90	1827.00	55.56
1852.13	70.09	1831.03	311.52
1856.86	301.13	1836.32	28.97
1862.26	110.93	1837.55	154.74
1863.49	563.51	1839.10	115.91
1868.07	3491.17	1852.89	117.15
1884.35	514.01	1853.93	106.84
1891.57	699.58	1856.20	802.50
1894.75	349.95	1863.96	1245.30
1902.48	1514.45	1872.03	1390.51
1912.72	3393.25	1879.16	904.03
1919.41	1744.19	1884.41	1224.22
1931.85	3112.86	1896.88	642.74
1970.11	262.66	1916.75	1634.83
1980.68	206.45	1964.79	227.62
1989.06	207.89	1981.79	464.34
1993.42	236.43	1982.64	23.85
1998.37	740.19	1983.85	46.12
2002.46	412.70	1985.93	179.48
2003.88	451.05	1993.81	200.54
2006.47	148.71	1997.87	450.87
2011.91	690.48	1999.80	277.77
2023.99	1367.32	2005.75	844.57
2026.93	2078.93	2007.92	497.41
2044.89	3582.04	2058.68	7387.40
2050.55	13212.50	2074.21	7959.70
2058.48	13178.79	2079.73	7060.52
2092.24	1600.59	2103.97	93.38

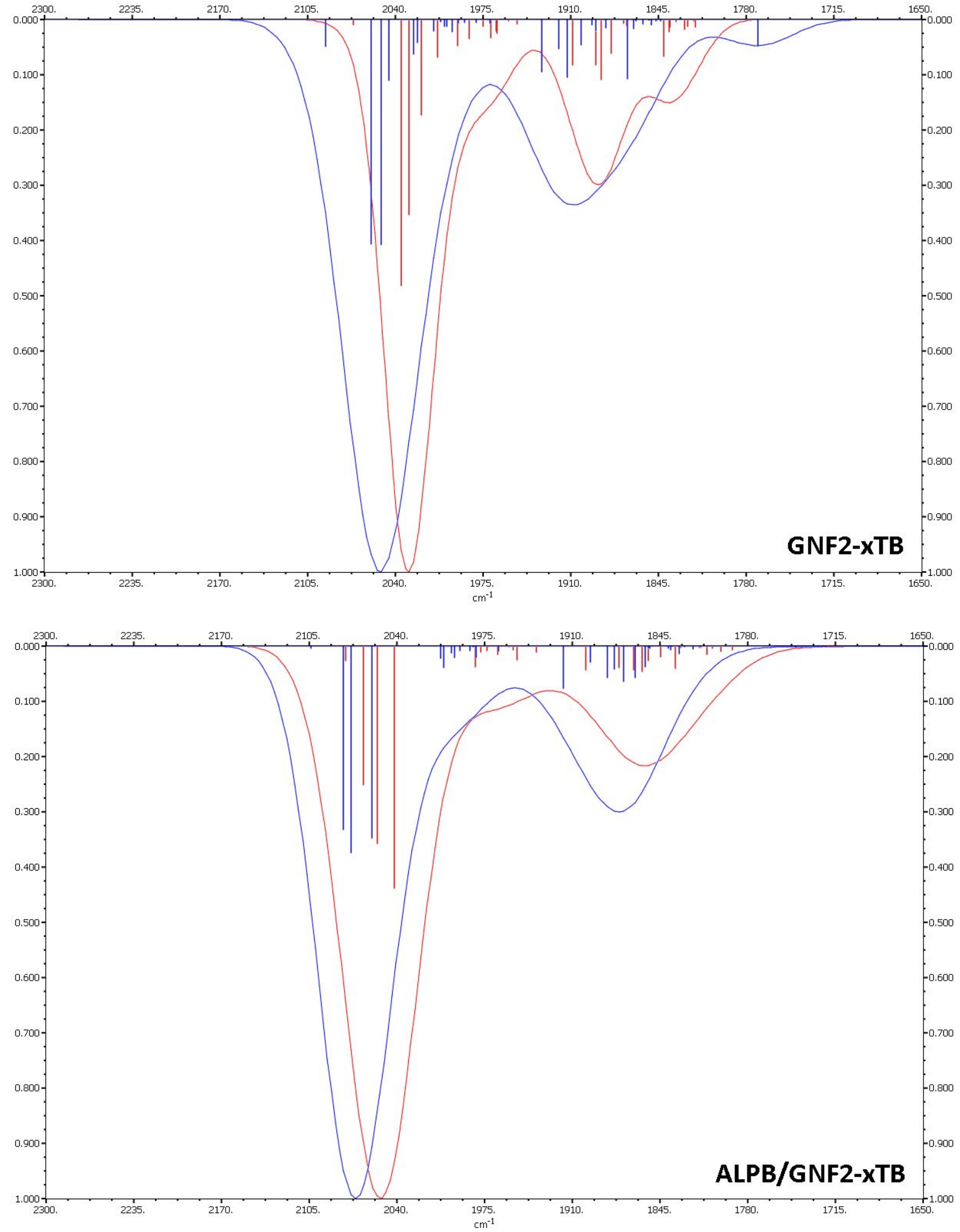

Figure S4. Simulated IR spectra of **1**$^{4-}$ (red) and **1**$^{3-}$ (blue) at GFN2-xTB and ALPB/GNF2-xTB levels.

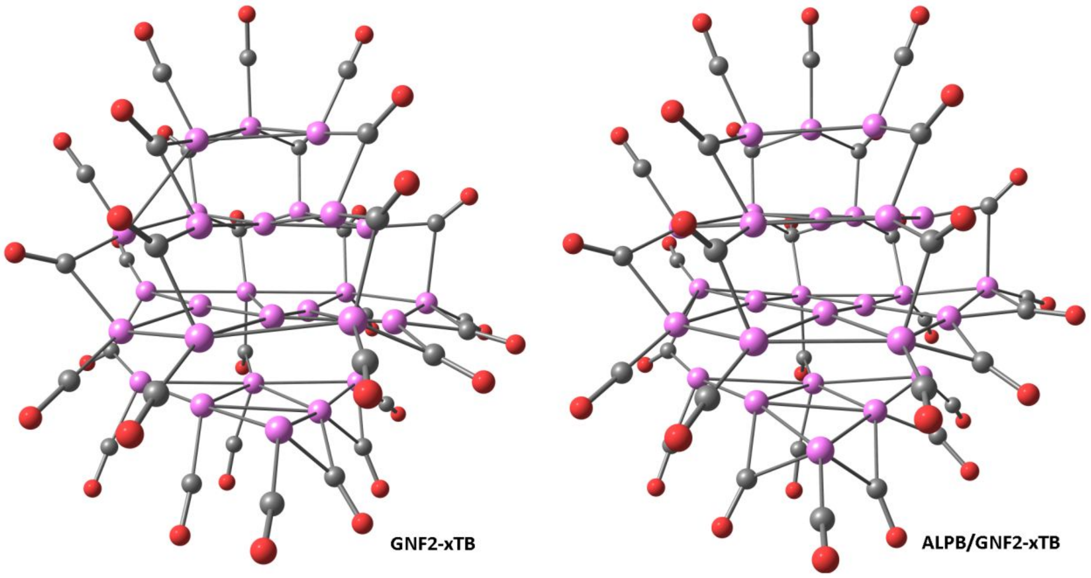

Figure S5. Computationally optimized structures of $\mathbf{1}^{3-}$ (purple, Pt; red, O; grey, C). The Pt-Pt interactions among the layers were not drawn for clarity.

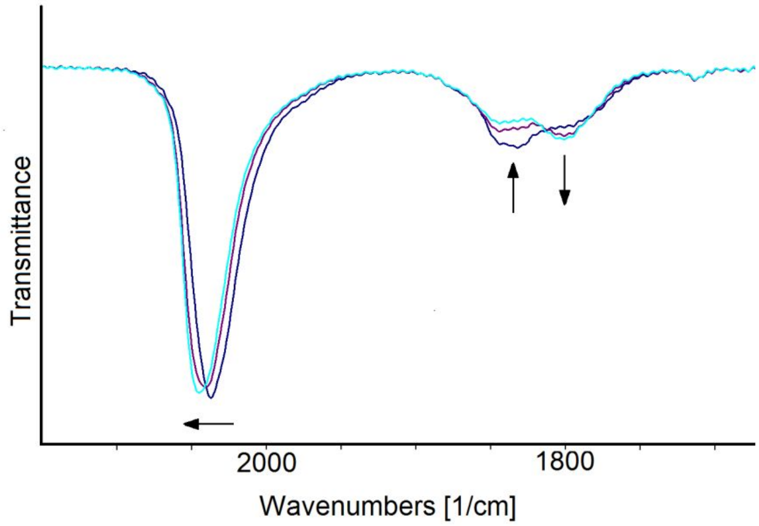

Figure S6. IR spectral changes of a CH_3CN solution of **1**$^{4-}$ recorded in an OTTLE cell to the potential of +0.24 V (blue line), +0.30 V (violet line) and +0.24 V (turquoise line) (*vs* Ag pseudo-reference electrode) during the slow cyclic voltammetry (scan rate 1 mV sec^{-1}) between –0.24 and +0.30 V. [N^{n}Bu$_4$][PF$_6$] (0.1 mol dm^{-3}) as the supporting electrolyte. The absorptions of the solvent and supporting electrolyte have been subtracted.

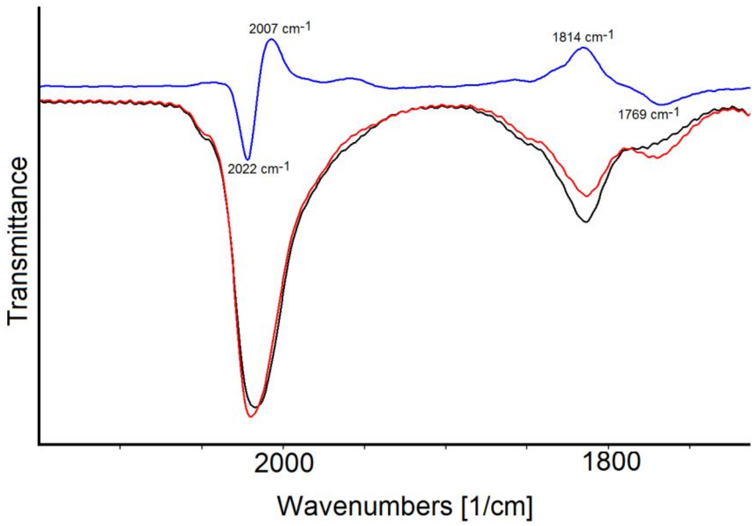

Figure S7. IR spectra of a CH_3CN solution of **1**$^{4-}$ recorded in an OTTLE cell before (black line) and after (red line) a cyclic voltammetry between–0.24 and +0.30 V *vs* Ag pseudo reference electrode (scan rate 1 mV sec^{-1}). The difference spectrum (red – black) is reported in blue. [N^{n}Bu$_4$][PF$_6$] (0.1 mol dm^{-3}) as the supporting electrolyte. The absorptions of the solvent and supporting electrolyte have been subtracted.

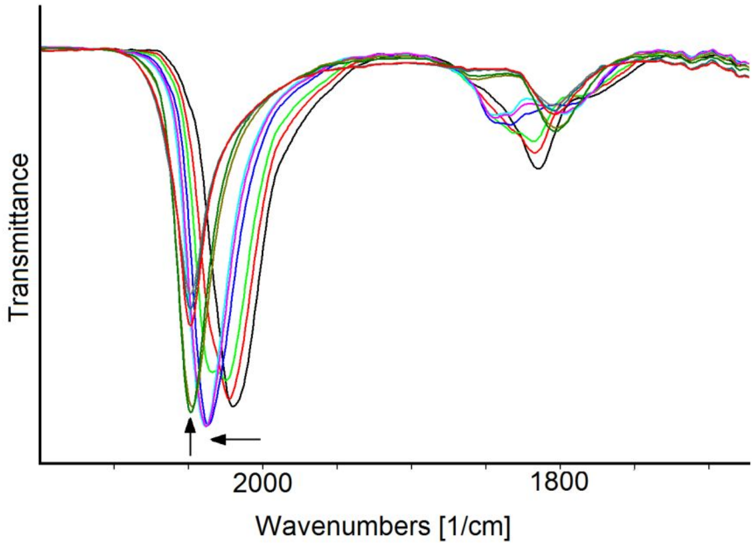

Figure S8. IR spectra of a CH_3CN solution of **1**$^{4-}$ recorded in an OTTLE cell during the progressive increase of the potential from –0.24 to +0.80 V *vs* Ag pseudo-reference electrode (scan rate 2 mV sec^{-1}). $[N^nBu_4][PF_6]$ (0.1 mol dm^{-3}) as the supporting electrolyte. The absorptions of the solvent and supporting electrolyte have been subtracted.

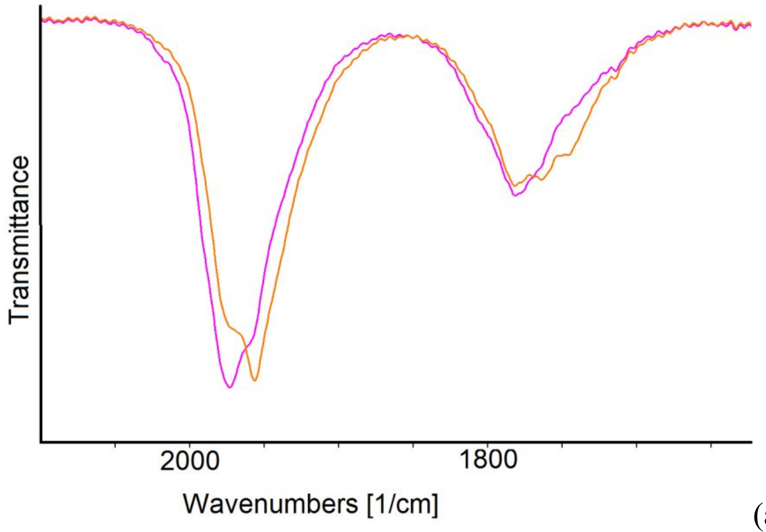

(a)

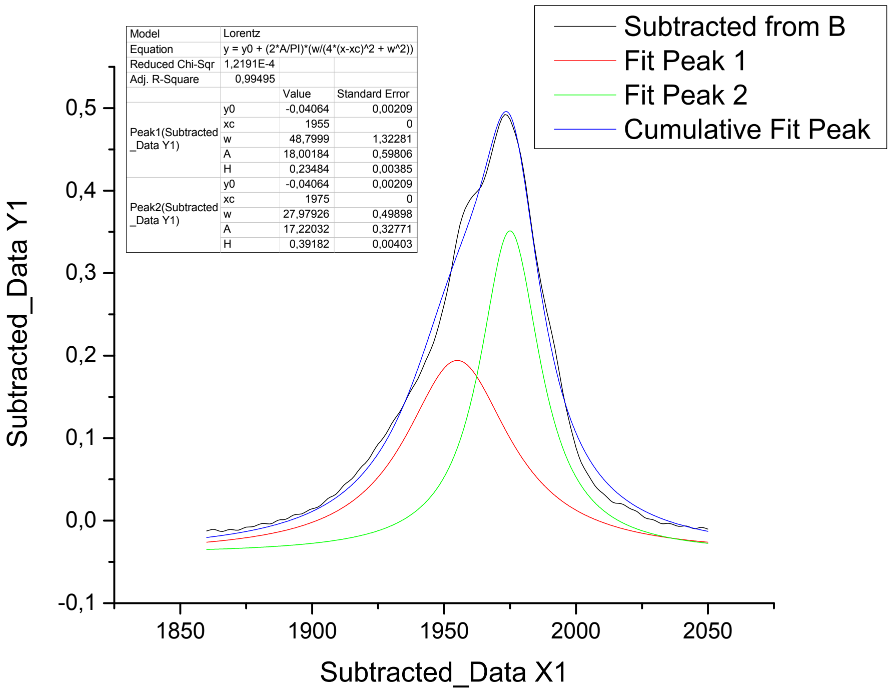

(b)

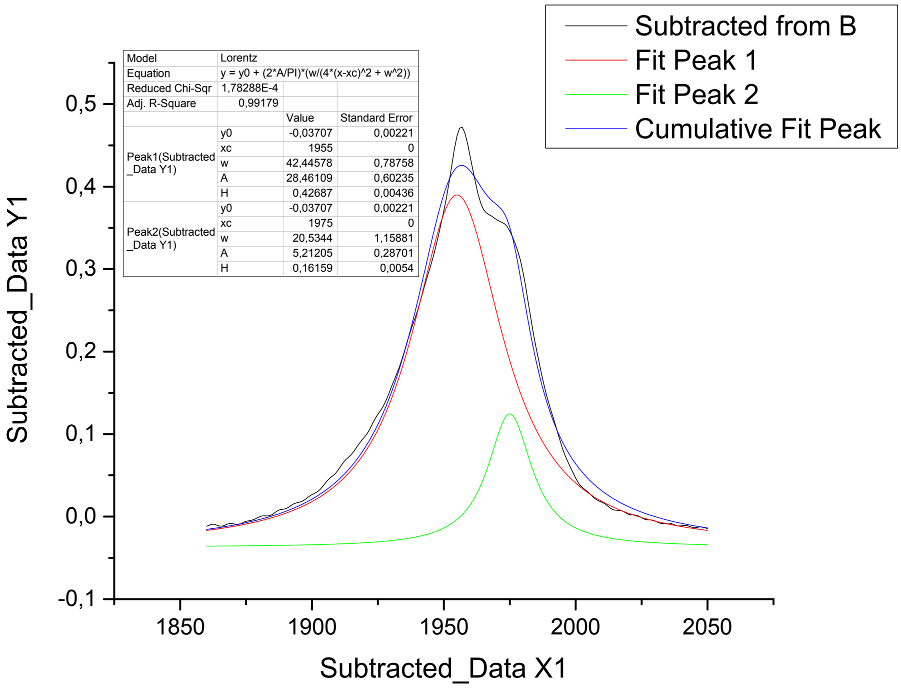

(c)

Figure S9. (a) Consecutive IR spectra of a CH_3CN solution of **1**$^{4-}$ acquired in an OTTLE cell to the potential of –1.74 V (fuchsia line) and –1.80 V (orange line) (*vs* Ag pseudo-reference electrode) during the slow cyclic voltammetry (scan rate 1 mV sec^{-1}) between –1.44 and –2.0 V (Figure 10(c)). Their spectral deconvolution is reported in (b) and (c), respectively, and shows the relative contribution to the overall spectra of two bands at 1975 and 1955 cm^{-1} (green and red curves, respectively), in different ratios according to the potential scan direction.
